# Pediatric Emergency Medicine Didactics and Simulation (PEMDAS): Pediatric Diabetic Ketoacidosis

**DOI:** 10.15766/mep_2374-8265.11098

**Published:** 2021-02-17

**Authors:** Cale Roberts, Ashley Keilman, Jean Pearce, Alissa Roberts, Kevin Ching, Jenny Kingsley, Alexander Stephan, Isabel Gross, Daisy Ciener, Julie Augenstein, Anita Thomas

**Affiliations:** 1 Pediatrics Resident, Department of Pediatrics, University of Washington School of Medicine and Seattle Children's Hospital; 2 Assistant Professor, Department of Pediatrics, Division of Emergency Medicine, University of Washington School of Medicine and Seattle Children's Hospital; 3 Assistant Professor, Department of Pediatrics, Division of Emergency Medicine, Medical College of Wisconsin; 4 Assistant Professor, Department of Pediatrics, Division of Endocrinology and Diabetes, University of Washington School of Medicine and Seattle Children's Hospital; 5 Associate Professor of Clinical Pediatrics, Division of Emergency Medicine, Weill-Cornell Medicine; 6 Assistant Professor, Department of Anesthesia and Critical Care Medicine, Division of Pediatric Critical Care, Keck School of Medicine, University of Southern California and Children's Hospital of Los Angeles; 7 Assistant Professor, Department of Emergency Medicine, Division of Pediatric Emergency Medicine, New York Presbyterian/Weill Cornell Medical Center; 8 Assistant Professor of Pediatrics, Department of Pediatric Emergency Medicine, Yale University School of Medicine; 9 Program Director of Pediatric Emergency Medicine Fellowship and Assistant Professor of Clinical Pediatrics, Division of Pediatric Emergency Medicine, Vanderbilt University Medical Center; 10 Base Hospital Medical Director, Quality and Safety Medical Director, and Attending Physician, Phoenix Children's Hospital; Clinical Assistant Professor of Child Health and Emergency Medicine, Mayo Clinic College of Medicine and Science and University of Arizona College of Medicine; 11 Director of Pediatric Emergency Medicine Fellow Simulation and Assistant Professor, Department of Pediatrics, Division of Emergency Medicine, University of Washington School of Medicine and Seattle Children's Hospital

**Keywords:** Pediatric Emergency Medicine, Diabetic Ketoacidosis, Simulation, Pediatric Critical Care Medicine, Pediatric Endocrinology

## Abstract

**Introduction:**

Diabetic ketoacidosis (DKA) is a life-threatening illness which classically presents with polyuria, polydipsia, and polyphagia that can rapidly progress to severe dehydration and altered mental status from cerebral edema. Younger patients may present with subtle or atypical symptoms that are critical to recognize and emergently act upon. Such patients are often cared for by teams in the emergency department (ED) requiring multidisciplinary collaboration.

**Methods:**

This simulation case was designed for pediatric emergency medicine fellows and residents. The case was a 14-month-old male who presented to the ED with respiratory distress and dehydration. The team was required to perform an assessment, manage airway, breathing and circulation, and recognize and initiate treatment for DKA including judicious fluid administration and an insulin infusion. The patient developed altered mental status with signs of cerebral edema requiring the initiation of cerebral protection strategies. We created a debriefing guide and a participant evaluation form.

**Results:**

Forty-two participants completed this simulation across seven institutions including attendings, residents, fellows, and nurses. The scenario was rated by participants on a 5-point Likert scale and was generally well received (*M* = 5.0). Participants rated the simulation case as effective in teaching how to recognize (*M* = 4.8) and manage (*M* = 4.5) DKA with cerebral edema in a pediatric patient.

**Discussion:**

This simulation represents a resource for learners in the pediatric ED in the recognition and management of a toddler with DKA and can be adapted to learners at all levels and tailored to various learning environments.

## Educational Objectives

By the end of this activity, learners will be able to:

1. Demonstrate the ability to assess and emergently manage airway, breathing, circulation and disability in a pediatric toddler-aged patient with vomiting and irritability, including frequent reassessments.
2. Verbally identify diabetic ketoacidosis (DKA) in a child.
3. Verbalize an appropriate differential diagnosis for this patient presentation.
4. Demonstrate appropriate fluid and electrolyte management in pediatric DKA.
5. Verbalize the risk and signs/symptoms of cerebral edema in pediatric DKA.
6. Utilize effective team leadership, roles, and communication.

## Introduction

Prompt identification of diabetic ketoacidosis (DKA) can be particularly difficult in toddlers as symptoms are often nonspecific, and diaper use can make classic polyuria and nocturnal enuresis less noticeable.^1^ DKA is a common acute complication at the time of diagnosis of diabetes mellitus, occurring in approximately one-third of type 1 diabetes new onset cases.^2^ Children under 3 years of age are at increased risk of DKA at time of diagnosis of diabetes mellitus compared to older children,^3^ complicated by challenges in diagnosing DKA in a nonverbal young child who is presenting with what might seem like a common childhood upper respiratory illness or acute gastroenteritis. The purpose of this simulation- based curriculum was to enhance the recognition and management of DKA in young patients.

DKA occurs when serum insulin levels are insufficient in relation to elevated counterregulatory hormones resulting in the triad of hyperglycemia (blood glucose > 200 mg/dL), ketonemia (serum b-hydroxybutyrate ≥ 3 mmol/L) or ketonuria (≥2+, moderate or large), and acidemia (venous pH < 7.3 or serum bicarbonate level < 15 mmol/L).^4^ Complications of DKA include osmotic diuresis causing dehydration and electrolyte wasting, metabolic and lactic acidosis, cerebral edema, and potentially death.^4^

Pediatric DKA is managed differently than DKA in adult patients, and it is critical that health care providers that care for pediatric patients with DKA be well versed in appropriate management. DKA treatment should begin with management of airway, breathing, and circulation in conjunction with Pediatric Advanced Life Support guidelines. The overall goals of treatment are aimed at treating dehydration (typically 5%–10% extracellular fluid volume deficit^5^) and acidosis, as well as reversing ketosis. One of the most feared complications of pediatric DKA is cerebral edema. Cerebral edema occurs in approximately 1% of patients with DKA, however it has been reported to contribute to 30%–50% of the morbidity related to DKA.^6^ Thus, this is a high risk, low occurrence phenomenon that can be seen in DKA progression. The pathophysiology of its development is not well understood. Patients should undergo frequent neurologic assessments for signs of cerebral edema (i.e., headaches, vomiting, altered mental status, pupillary abnormalities, and Cushing's Triad). If signs or symptoms of cerebral edema develop, treatment should begin immediately and include neuroprotective measures such as elevating the head of bed, administration of 3% hypertonic saline or mannitol, and avoidance of hypotension, hypoxia, and excessive fluid administration.^4^ Health care providers who care for pediatric patients with DKA should be proficient in these maneuvers when caring for cerebral edema related to DKA as it carries high morbidity.^6^ Additionally, as initial presentation of type 1 diabetes as DKA in a toddler can be challenging to diagnose, it is important to consider this diagnosis and be well versed in management as improper management can be dangerous. Therefore, simulation is an appropriate medium for practicing management of pediatric DKA with progression to cerebral edema, particularly as management of adult patients with DKA differs.

This curriculum was developed for pediatric emergency medicine (PEM) fellows, and pediatric and emergency medicine residents, but could be adapted for interdisciplinary groups including fellows, residents, medical students, nurses, advanced practice providers, pharmacists, and respiratory therapists. In some institutions, attending physicians may participate alongside trainees and ancillary staff to reinforce their skills or ensure sufficient participants for a simulation session; when this occurs, attendings typically allow trainees to take the lead in managing simulated patients. The management of pediatric DKA is not limited to physicians or physician trainees and can be approached from an interprofessional perspective. This simulation curriculum can be used in conjunction with other PEM simulation curriculum on *MedEdPORTAL*^7–29^ or as a standalone curriculum.

## Methods

PEM physicians with simulation and curricular development experience developed this simulation scenario (Appendix A) in conjunction with a pediatric endocrinologist to be utilized across the PEM didactics and simulation (PEMDAS) network for PEM fellows and residents who might rotate in the pediatric emergency department (ED) (pediatrics/emergency medicine). There was prereading available in Appendix A, however, across the PEMDAS network this was not provided in order to preserve the element of surprise with the simulation case. The scenario was based on an actual clinical case and adjusted to meet educational objectives.

Appropriate equipment and environment preparation material was provided (Appendix B). Participants had experience with the diagnosis and emergency management of pediatric patients presenting with classic new onset type 1 diabetes mellitus symptoms and DKA prior to the simulation.

The critical actions checklist (Appendix C) was created by the facilitators with the oversight of author Alissa Roberts, MD as the pediatric endocrinologist content expert through a modified Delphi process to ensure that it was in alignment with the educational objectives of the simulation scenario. The scenario was facilitated by experienced PEM simulation faculty across seven institutions who provided content expertise and constructive feedback to learners with regards to the learning objectives. Participants obtained a focused history, completed a patient assessment, developed a differential diagnosis for irritability and vomiting in a toddler in the setting of lab values diagnostic of DKA, initiated fluid resuscitation and insulin infusion, verbalized and responded appropriately to worsening mental status, and determined an appropriate patient disposition. Supplemental material including lab values, an ECG, and a chest X-ray were provided (Appendix D).

Debriefing was conducted immediately following the simulation using the PEARLS^30^ method as the debriefing framework. Standard debriefing materials were developed specific to this case (Appendix E) as well as a teamwork and communication guide (Appendix F). Appendix F utilizes TeamSTEPPS terminology, which all experienced simulation facilitators should be familiar with as it is standard within simulation; however, definitions were provided for novice facilitators to review with participants when debriefing and to utilize when facilitating the simulation. A didactic slides-based presentation (Appendix G) was created with the help of a pediatric endocrinologist content expert (author Alissa Roberts, MD) for distribution in advance of the simulation for less experienced learners or following the simulation for more experienced learners. In all iterations across the PEMDAS network, Appendix G was utilized during the debriefing session as an adjunct as opposed to providing it ahead of the simulation. We asked all participants to complete an evaluation (Appendix H) following the debrief sessions. We used the feedback from this evaluation to hone the simulation in subsequent iterations.

### Equipment/Environment

The simulation was conducted in either an actual ED room or an ED room in a simulation center using a high-fidelity infant manikin (Laerdal SimBaby). When an additional facilitator was available, they acted as the parent. We developed a detailed equipment list for preparation of the simulation environment (Appendix B). The scenario started with the patient without IV access and was not on monitors. We provided the participants with simulated medications, IV fluids, and other equipment commonly available in a pediatric ED setting. Throughout the scenario the facilitator provided laboratory and imaging results as requested by participants including a chest X-ray, lab values, and ECG (Appendix D).

### Personnel

Each simulation session accommodated up to 10 participants as outlined in Appendix A, including up to two facilitators. When the scenario was conducted with a smaller team, a second facilitator functioned as a bedside nurse to place the patient on monitors, obtain IV access, draw labs, and administer medications. When the scenario was conducted with multidisciplinary teams, participants remained in roles consistent with their typical clinical role to maximize the fidelity and learning experience.

### Implementation

This case was targeted towards medical personnel who work in a pediatric ED and it was expected that most learners had some familiarity with the recognition and management of new onset DKA in an emergency setting. The scenario began with a parent bringing in a 14-month-old toddler with fussiness, emesis, and difficulty breathing. At the start of the case the registered nurse (RN) called in the team with concern for dehydration and increased work of breathing. The RN had already initiated a 20 mL/kg normal saline bolus. An embedded participant or the facilitator played the role of the parent who provided a brief history that the patient has had 3 days of fussiness, nasal congestion, and emesis. Additional history was available at participant request (Appendix A). After a team member attached monitors, the vital signs were provided to the care team via a simulated monitor. The patient appeared dehydrated and diaphoretic requiring fluid resuscitation. Upon team request, lab work was provided showing results consistent with DKA. The participants initiated treatment for DKA including judicious intravenous fluid administration and an insulin infusion. The patient ultimately developed altered mental status, bradycardia, and hypertension related to cerebral edema secondary to DKA. This necessitated treatment of increased cranial pressure (elevation of the head of the bed, administration of hypertonic saline or mannitol, patient hyperventilation, and involvement of neurosurgery). At participant request, an ECG (Appendix D) was available and notable for sinus tachycardia and the chest X-ray (Appendix D) was unremarkable. Appropriate sign-out to the pediatric ICU was expected at the conclusion of the scenario. Appendix E provided a debriefing framework, Appendix F provided a glossary of terminology for teamwork and communication, Appendix G provided a slide-based didactic, and Appendix H was the evaluation form for participants to complete after the case.

### Debriefing

We used the PEARLS^30^ debriefing method and the tools in Appendices E and F to assist in facilitating debrief sessions after the simulations. The debriefing guide provided an outline for facilitators leading a debriefing session based on the participant pool. We recommended that debriefs begin by allowing participants to share their overall reflection of the scenario, followed by a more structured discussion as outlined in Appendix E. Participant impressions were then used to guide the conversation and provide segues into teamwork and communication (Appendix F) as well as reiterate diagnostic and management skills. For the more experienced debrief facilitator, a quick reference to high-yield debriefing medical points about DKA was created and added to Appendix E based on feedback from facilitators across PEMDAS sites.

### Assessment

The scenario was facilitated and debriefed by PEM physicians, who were also experienced in simulation. Facilitators provided pediatric resuscitation and content expertise as well as participant performance feedback in accordance with the learning objectives. Following scenario debriefing, participants completed the evaluation form (Appendix H) to give facilitators feedback on the relevance, realism, and overall learning experience of the simulation scenario by responding to Likert scale evaluative statements (1 = *strongly disagree*, 2 = *disagree*, 3 = *neutral*, 4 = *agree*, 5 = *strongly agree*). Participants also had the opportunity to provide additional feedback on the clinical impact and ideas for scenario improvement with free response questions. Surveys were analyzed to aid with scenario improvement for subsequent iterations.

## Results

Surveys were voluntarily completed by all 42 participants across seven institutions, a 100% (42 of 42) survey response rate. Of the 42 participants, 27 identified as PEM fellows, five as pediatrics residents, four as emergency medicine residents, one as an attending, one as a medicine/pediatrics residents, and four did not identify their roles; however, it was reported that nurses participated in at least one site where surveys were filled out without roles identified, thus some of those evaluations may have been filled out by nursing participants.

The scenario was generally well received and highly rated on average for being relevant to their work (median and *M* = 5.0; Table 1). In addition, participants rated the simulation case as effective in teaching how to recognize (*M* = 4.8) and manage (*M* = 4.5) DKA with cerebral edema in a pediatric patient.

**Table 1.**
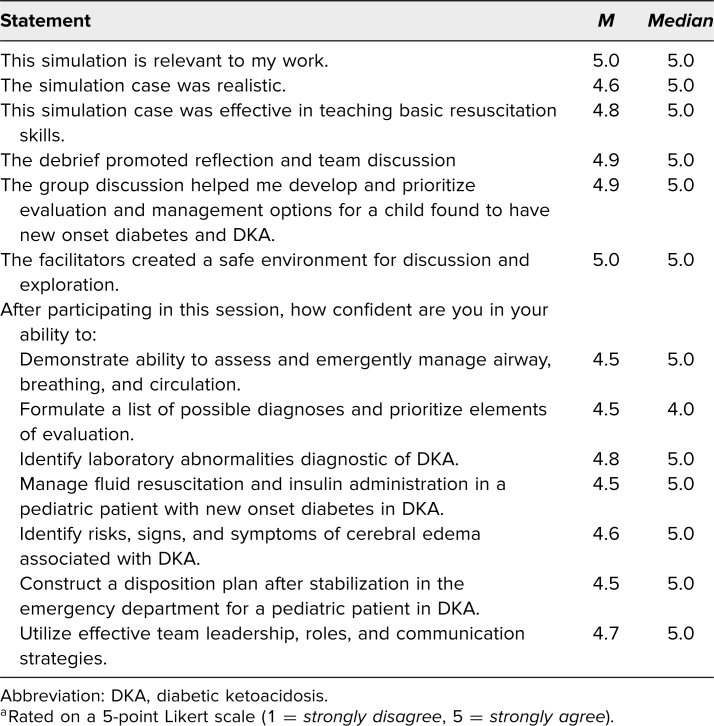
Cumulative Evaluation Scores (*N* = 42)^a^

In response to the open-ended self-reported knowledge question “Can you list/describe one or more ways this simulation session will change how you do your job?” participant comments fell into the following themes: expansion of differential diagnosis, importance of early blood glucose checks, importance of fluid and insulin management in DKA, and management of cerebral edema (Table 2). In response to the free-response question on suggested improvements to the scenario, participant responses fell into the following themes: changes to simulation environment, no changes, increase difficulty of simulation, increase length of simulation (Table 3). General participant comments included: “Awesome sim—realistic, helpful,” “Well written case, easy to follow,” “Awesome sim, great debrief, we learned so much,” and “Great case, good learning clinically and team structure/communication.”

**Table 2.**
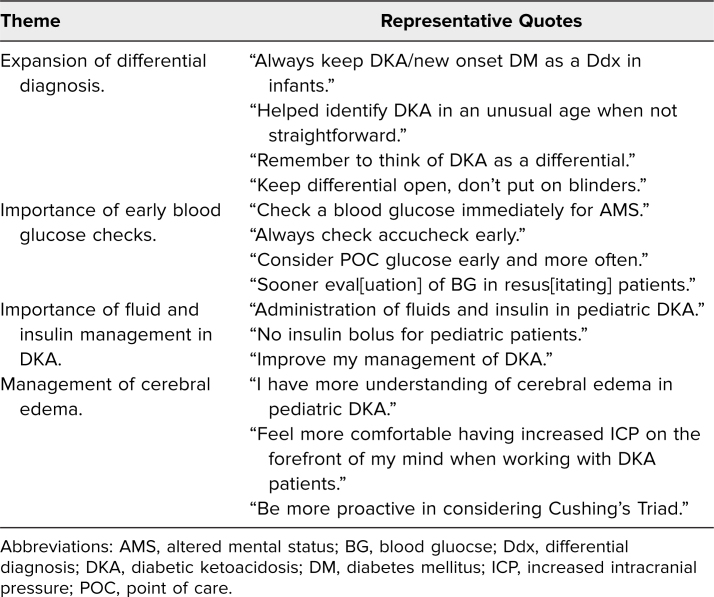
Themes Obtained From Self-Reported Knowledge Question: “Can You List/Describe One or More Ways This Simulation Session Will Change How You do Your Job?”

**Table 3.**
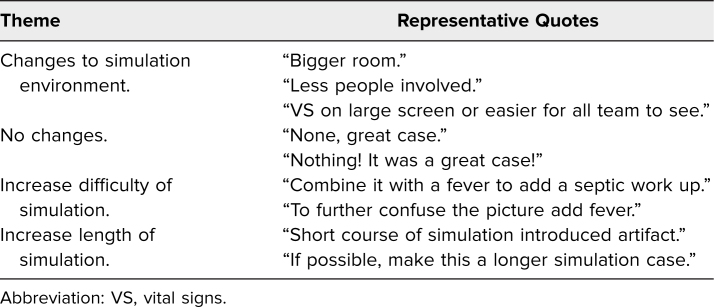
Themes Obtained From the Free-Response Question: “How Could we Improve This Scenario?”

## Discussion

The purpose of this curriculum was to provide an environment to aid learners in the recognition and management of pediatric DKA. The curriculum was designed to allow providers to review fluid and insulin management in pediatric DKA, along with the recognition and management of cerebral edema, a serious complication in pediatric DKA. Given that many younger patients do not present with classic symptoms of DKA, this curriculum provided an opportunity for learners to expand their differential diagnosis to include DKA in younger pediatric patients presenting with respiratory distress and vomiting leading to dehydration. For many pediatric and emergency medicine providers, this scenario is not uncommon and thus prompt recognition and appropriate management of pediatric DKA is crucial. Furthermore, this scenario allowed learners to work on teamwork and communication skills in a controlled environment. It also provided a dedicated space to debrief regarding team dynamics.

Kirkpatrick's model level 1 of reaction^31^ was targeted in the development of this simulation curriculum, particularly as evaluations (Appendix H) were designed to be completed immediately after the debriefing. Reactions were also captured during the debriefing by participants verbally providing feedback. Verbal and written reactions were subsequently used in other iterations of the curriculum and also allowed facilitators to gain a sense of the team's knowledge of pediatric DKA. Thus, some elements of Kirkpatrick's level 2 of learning^31^ were elucidated in the debriefing portion when discussing the case learning points. Additionally, simulation instructors who have undergone formal simulation facilitator training often have learners state one piece of learning from the case as a method of concluding the debriefing. Users of this curriculum should bear in mind that this simulation was designed to be more formative rather than summative in terms of evaluating the team's knowledge and teamwork.

Overall, this simulation was well received by learners. Most participants felt that the case was relevant to their work (median and *M* of 5). Learners indicated that they felt confident in the diagnosis and management of DKA as well (Table 1). We felt that it was successful because we were able to run the scenario at various sites. Piloting this scenario was necessary to refine the material and adapt to learners of different levels. We discovered that some participants felt the scenario was too short and wanted airway practice, and so intubation was added as a step. Those participants who incorporated intubation also requested a pre/post intubation checklist specific to those institutions and so this was added to those specific sites. Other sites kept the scenario as is without intubation. Other sites preferentially utilized mannitol or hypertonic saline in treatment of cerebral edema, so we suggest that sites inquire as to their preferred or most available agent of choice and utilize that. Additionally, at some sites, pediatric intensive care and other subspecialists are involved in the care of these patients much earlier and so we recommend that this be tailored to specific sites. Participants at one site requested making the lead-in more complicated (i.e., add fever to the patient's presentation to broaden the differential diagnosis to sepsis); however, we did not adapt this at all sites, as feedback from facilitators indicated that the case may have veered down a different management pathway and distracted from our learning objectives. However, given that it may be more difficult to identify DKA as the culprit, we suggest that fever can be piloted with more advanced learners, such as PEM fellows and attendings, though we would recommend that more time be allotted for both the scenario and debriefing, along with well placed embedded participants to help steer team members if needed.

In some institutions, nurses do not order fluids prior to a physician evaluation and thus the lead-in of the bedside nurse initiating fluids was not realistic. Some institutions opted to change this such that no fluids were started, and others had the patient transferred from an outside facility with fluids already running. We recommend that each facility execute the simulation to adhere to reality as much as possible in order to translate the simulation learning to real practice.

Lastly, some facilitators found the extensive debriefing guide to be quite long, and so one site distilled it to a one-page high-yield of debriefing points, which was added to Appendix E and targeted towards the more experienced debrief facilitator, and can also be utilized if lack of time is an issue in debriefing.

### Limitations

Limitations in this simulation curriculum included that it was tested on PEM fellows, a PEM attending, pediatric residents, emergency medicine residents, and medicine-pediatrics residents, with four participants not declaring their roles. This case is applicable to any health care provider who would provide care to a pediatric patient with DKA who did not specifically participate in our simulation, including attending general pediatric physicians, attending emergency medicine physicians, medical students, nursing students, respiratory therapists, pharmacists, and midlevel providers. An additional limitation was that not all of our participants indicated their roles on the evaluation (Appendix H). Manikin limitations included not being able to accurately portray capillary refill and perfusion status, though this may be mitigated by higher fidelity models, or by providing a video of a patient's delayed capillary refill, or by the facilitator providing an assessment on the patient's capillary refill when prompted. Additionally, some learning objectives were more subjective in nature, such as teamwork and communication, and were not conducive to objective measurement with this curriculum. This curriculum was assessed primarily via Kirkpatrick's level 1,^31^ as mentioned in the discussion, but did not delve as much into learning or Kirkpatrick's level 2. In future trials, performance could be measured more formally by repeating it with the same group of learners multiple times over a prolonged time period. For this curriculum, as we used a convenience sample of learners, it was not feasible to perform pre- and posttesting on all learners to assess performance/knowledge. Additionally, while the PEMDAS group encompasses institutions across the United States, we did use a convenience sample of providers, thus potentially limiting generalizability. Lastly, translation of knowledge acquired from this session to actual clinical resuscitations was not measured by our evaluation tool.

### Implications

The simulation trials at various institutions provided us with ways in which the scenario could be improved. Some participants felt the simulation was too short. To increase the length of the simulation, facilitators could have learners intubate the patient once they develop cerebral edema. Our results also highlighted the need to adapt the simulation to be more standard with the local practice including things like team size and availability of medications on hand (e.g., hypertonic saline vs. mannitol). Facilitators should take what is standard for their clinical environment into account when preparing the scenario.

## Appendices

Ped DKA Simulation Case.docxPed DKA Environmental Preparation.docxPed DKA Critical Actions.docxPed DKA ECG CXR Labs.docxPed DKA Debriefing Materials.docxPed DKA TeamSTEPPS Glossary.docxPed DKA Slides.pptxPed DKA Evaluation Form.docx
All appendices are peer reviewed as integral parts of the Original Publication.
