# Supplementary material for: Pediatric Emergency Medicine Didactics and Simulation (PEMDAS): Pediatric Diabetic Ketoacidosis
Source: MedEdPORTAL. 2021 Feb 17;17:11098. doi: 10.15766/mep_2374-8265.11098 (PMC7901255; doi:10.15766/mep_2374-8265.11098)
Supplement: Supplementary file 1 — Ped DKA Simulation Case.docxPed DKA Environmental Preparation.docxPed DKA Critical Actions.docxPed DKA ECG CXR Labs.docxPed DKA Debriefing Materials.docxPed DKA TeamSTEPPS Glossary.docxPed DKA Slides.pptxPed DKA Evaluation Form.docx [file mep_2374-8265.11098-s001.zip › A. Ped DKA Simulation Case.docx]

| **Appendix A: MedEdPORTAL Simulation Case Template**  **SIMULATION CASE TITLE: Pediatric Emergency Medicine Didactics and Simulation (PEMDAS): Pediatric Diabetic Ketoacidosis**    **Authors: Cale Roberts MD; Ashley Keilman, MD; Jean Pearce, MD, MS; Alissa Roberts, MD; Kevin Ching, MD; Jenny Kingsley, MD; Alexander Stephan, MD; Isabel Gross, MD, PhD, MPH; Daisy Ciener, MD, MS; Julie Augenstein, MD; Anita Thomas, MD, MPH** | |
| --- | --- |
| **PATIENT NAME: Julian**  **PATIENT AGE: 14 months**  **CHIEF COMPLAINT: Emesis** | |
|  | |
| **Brief narrative description of case** | Julian is a 14-month old male presenting with 1 day of nasal congestion, non-bloody non-bilious (NBNB) emesis and intermittent episodes of fussiness without diarrhea, fever or decreased urine output. Upon arrival to the ED he is noted to be tachypneic, fussy and clinically dehydrated. On further history, the patient has been noted to have an unsteady gait compared to baseline, increased urine output for the past 3 days and a 1-2 lb weight loss. A bedside nurse (RN) starts a fluid bolus for clinical dehydration. Anticipated initial interventions include obtaining a point-of-care (POC) glucose, urinalysis and additional laboratory tests to confirm diagnosis of DKA. The team should initiate gradual fluid resuscitation and an insulin infusion. Endocrinology and Pediatric Intensive Care Unit (PICU) consultations should be obtained. The patient will become more tachycardic, continue to have (NBNB emesis and frequent urine output. He will develop increasingly altered mental status and become obtunded if given excess fluid resuscitation, or insulin or bicarbonate boluses.  Throughout the case participants will need to frequently reassess the patient to determine the effectiveness of their interventions. The case is completed with admission to the PICU. |
| **Primary Learning Objectives** | 1. Demonstrate the ability to assess and emergently manage airway, breathing, circulation and disability in a pediatric toddler-aged patient with vomiting and irritability, including frequent reassessments. 2. Verbally identify diabetic ketoacidosis (DKA) in a child 3. Formulate an appropriate differential diagnosis for this patient presentation. 4. Demonstrate appropriate fluid and electrolyte management in pediatric DKA. 5. Identify the risk and signs/symptoms of cerebral edema in pediatric DKA. 6. Utilize effective team leadership, roles, and communication. |
| **Critical Actions** | State #1: Presentation   1. Complete primary and secondary patient assessments    1. Place the patient on monitors    2. Establish two working vascular access points    3. Obtain and interpret diagnostic studies:       1. Labs: venous blood gas (VBG), POC glucose, electrolytes, urine ketones (or serum ketones if available) 2. Collect a focused history 3. Develop a differential diagnosis for irritability and vomiting in a toddler in the setting of lab values suggestive of DKA 4. Begin treatment with isotonic fluid rehydration and insulin administration    1. Start with a 20 ml/kg rapid normal saline (NS) bolus and adjust additional fluids accordingly     Clinical State #2: Worsening Agitation and Vomiting   1. Identify declining mental status in the setting of vomiting and worsening dehydration 2. Perform a repeat focused neurologic exam 3. Perform a clinical assessment of cerebral edema risk    1. Initiate maneuvers to reduce potentially increased intracranial pressure (ICP) (head of the bed elevation, administration of mannitol or hypertonic saline, patient hyperventilation) 4. Discuss airway support 5. Obtain a neurosurgical consultation 6. Discuss ordering or obtain a stat head CT   Clinical State #3: Improved Hemodynamics and Admission to Intensive Care Unit (ICU)   1. Transfer the patient to the ICU |
| **Learner Preparation** | - *Optional pre/post reading* - *If learners are unfamiliar with DKA they may be provided with pre-simulation readings to prime them for the scenario*   - Cashen K, Peterson T. Diabetic Ketoacidosis. *Pediatrics in Review.* 2019; 40 (8) 412-420; doi: <https://doi.org/10.1542/pir.2018-0231>   - Cooke D, Plotnick L. Management of Diabetic Ketoacidosis in Children and Adolescents. *Pediatrics in Review.* 2008; 29 (12) 431-436; doi: <https://doi.org/10.1542/pir.29-12-431>.   - Helman A. EM Cases: Pediatric DKA*. emDOCS.net*. Available [*http://www.emdocs.net/em-cases-pediatric-dka/*](http://www.emdocs.net/em-cases-pediatric-dka/). Published Oct 12, 2018. Accessed Nov 15, 2019.   - Koves I et al. Improving Care for Pediatric Diabetic Ketoacidosis. *Pediatrics.* 2014; 134 (3) e848-e856; doi: <https://doi.org/10.1542/peds.2013-3764> - *More experienced learners may receive an article as post-simulation reading to reinforce the knowledge from the scenario*   - Kupperman N et al. Clinical Trial of Fluid Infusion Rates for Pediatric Diabetic Ketoacidosis. *The New England Journal of Medicine*. 2018; 378:2275-228. doi: 10.1056/NEJMoa1716816   - DeCourcey DD, Steil GM, Wypij D, Agus MD. Increasing Use of Hypertonic Saline Over Mannitol in the Treatment of Symptomatic Cerebral Edema in Pediatric Diabetic Ketoacidosis. *Pediatric Critical Care Medicine.* 2013;14(7):694–700. doi: 10.1097/PCC.0b013e3182975cab. |

| **Initial Presentation** | | | |
| --- | --- | --- | --- |
| **Initial vital signs** | Temp 36.8, HR 160, RR 31, BP 100/53, Sats 100%, weight 10 kg or Broselow | | |
| **Overall Appearance** | Well developed, well nourished fussy male toddler with mild respiratory distress and tachypnea held by a parent. | | |
| **Actors and roles in the room at case start** | Parent - may be played by a facilitator or an embedded participant  Bedside nurse (RN) - optional - if not available, a facilitator may state that the RN obtained IV access, started a fluid bolus for dehydration and vomiting and called the team into the room due to tachypnea and increased work of breathing.  Team members:  Doctor #1: Team Leader  Doctor #2: Survey MD  Doctor #3: Airway Physician (optional, this role can be combined with #2)  Doctor #4: Family Correspondent (optional, this role can be combined with #1 or #2)  Nurse #1: Medication Administration RN (optional, this role can be played by a facilitator or be assumed by the initial bedside RN)  Nurse #2: Medication Preparation RN (optional)  Nurse #3: Documenting RN (optional)  Nurse #4: Circulating RN (optional)  Instructor #1: Simulation instructor and debriefer, If a 2^nd^ instructor is not available, the facilitator can play the role of the parent as well. This instructor may also play the role of PICU attending at the end of the scenario.  Instructor #2: If a 2^nd^ instructor is available, you may cast them as the embedded participant “parent,” also available to answer questions and assist with debrief. | | |
| **HPI** | 14-month old previously healthy fully immunized male presenting with nasal congestion, fussiness, non-bloody, non-bilious emesis and tachypnea without diarrhea or fever. He has decreased PO intake but is making wet diapers. The parents became concerned after multiple episodes of emesis and increased work of breathing prompting them to come to the ED.  SAMPLE history (if asked):  Signs/Symptoms - The patient has been noted to have an unsteady gait compared to baseline, increased urine output for the past 3 days and a 1-2 lb weight loss in ED compared to his 12-month well child visit weight.  Allergies: None  Meds: Acetaminophen as needed  PMH: None  Last intake: Milk 1 hour prior to arrival  Events preceding: As above  ROS: Otherwise negative except as noted above  Social history: Lives with his parents and grandmother. He is cared for by his grandmother during the day. | | |
| **Past Medical/Surgical History** | **Medications** | **Allergies** | **Family History** |
| None | Acetaminophen as needed | No Known Drug Allergies | Not significant |
| **Physical Examination** | | | |
| **General** | Well developed, well nourished, fussy but consolable at times when held by parents. Tachypneic with increased work of breathing | | |
| **HEENT** | Normocephalic and atraumatic, clear rhinorrhea, making tears | | |
| **Neck** | Supple, full range of motion | | |
| **Lungs** | Clear to auscultation bilaterally, no stridor, no wheeze. Tachypneic with diffuse and prominent subcostal retractions | | |
| **Cardiovascular** | Tachycardic, regular rhythm, no murmur, no gallop, extremity pulses are normal, no edema, capillary refill is 3-4 seconds | | |
| **Abdomen** | Soft, nondistended, patient pushes examiners’ hands away with exam and cries/guards diffusely, no masses | | |
| **Neurological** | Alert, PERRL 3mm-->2mm, tracks well with eyes, normal hand/eye coordination, moves all extremities well, normal tone, crying and holding onto parents | | |
| **Skin** | Warm, dry, no rashes | | |
| **GU** | Normal external male genitalia with testes descended bilaterally without pain | | |
| **Developmental** | Age appropriate | | |

| **Instructor Notes - Changes and CASE Branch Points**  *This section should be a list with detailed description of each step than may happen during the case. If medications are given, what is the response? Do changes occur at certain time points? Should the nurse or other participant prompt the learners at given points? Should new actors or participants enter, and when? Are there specific things the patient will say or do at given times? There are a few examples given, but it is expected that most cases will have many more changes and potential branch points..* | | |
| --- | --- | --- |
| **Intervention / Time point** | **Change in Case** | **Additional Information** |
| *RN notifies the team that she/he is concerned about this patient with increased work of breathing, is worried he is dehydrated and lets team know that she/he had the resident order a 20 ml/kg normal saline bolus (NS)*  *T:0 minutes* | *Exam as above. Learners should establish team roles, assess ABCs, apply monitors, and obtain IV/IO access.*  *Resident ordered prior to the team coming in and the RN initiated a 20 ml/kg NS bolus* | *A: patent, crying*  *B: tachypneic with deep diffuse retractions but lungs are clear to auscultation bilaterally*  *C: tachycardic, 2+ distal pulses, CR ~3-4 seconds*  *D: GCS 15, looks at examiners and holding onto parents, crying, moves all extremities well, normal tone, PERRL 3 mm-->2mm*  *E: feels sweaty* |
| *Participants request labs*  *T: 3 minutes* | *Lab results are given only for those requested by the learner* | *Glucose 451*  *VBGs: pH 7.11, pCO2 15, pO2 80, bicarbonate 4.9, base excess -24.7*  *Electrolytes: Sodium 132 (hyperglycemia corrected sodium 138 but only provide if asked for*  *Corrected Na = measured Na +(((serum glc-100)/100) x 1.6)), potassium 5.2, bicarbonate 5, ionized calcium 1.44, magnesium 1.5, phosphorus 3.2*  *Lactate 1.8*  *CBC: WBC 18, hemoglobin 15.7, hematocrit 47, platelets 216*  *Beta-hydroxybutyrate (serum ketones if available) 11.5 mmol/dL*  *Urinalysis: large ketones, 3+ glucose*  *BUN 20, creatinine 0.6* |
| *Participants verbalize a differential diagnosis list including DKA and initiate management of a pediatric patient in DKA*  *T: 5 minutes* |  |  |
| *Participants request a reassessment of fluid administration and verbalize a need to slow down fluid rate by administering a 10ml/kg NS bolus over 1 hour*  *T: 5 minutes* | *HR decreases to 150 from 160, CR is 3 seconds* | *If asked for repeat glucose, is 390* |
| Participants administer an insulin infusion at 0.025-0.1 units/kg/hr |  | *If asked for repeat glucose, is 390* |
| Participants initiate IVF following the bolus (using a 2-bag system), to replace volume deficit gradually over 48 hours. Fluids should include potassium (K) if < 3.5 mEq/L and if creatinine is normal. Fluids should not include K if hyperkalemic (K >5.5 mEq/L). |  | *Method for calculating IVF rate for this patient:*   1. *Calculate the patient’s fluid deficit assuming 7% dehydration* 2. *Add the fluid deficit to 48 hours of total maintenance IVF* 3. *Subtract the amount of fluid already received (include any boluses)* 4. *Administer that total over 48 hours (first 4 hours detailed below)*   *Hour 1-4: (early electrolyte adjustment/rehydration):*  *Bag 1: NS + 40 mEq K phosphate/K acetate*  *Bag 2: Dextrose 10% (D10)/NS + 40 mEq K phosphate/ K acetate to be used only if glucose <300 or if precipitously falls* |
| If participants administer > 20ml/kg in total fluid boluses | Patient becomes obtunded, hypertensive to 125/90, and bradycardic to 80 | *If the team does not recognize a change in clinical status the embedded participant or RN may state “The blood pressure is “125/90 and the HR is 80”* |
| If participants administer a sodium bicarbonate bolus | Patient becomes obtunded, hypertensive to 125/90, and bradycardic to 80 | Repeat serum bicarbonate is 15.  *If the team does not recognize a change in clinical status the embedded participant or RN may state “The blood pressure is “125/90 and the HR is 80”* |
| If participants administer an insulin bolus | Patient becomes unresponsive, obtunded, hypertensive to 125/90, and bradycardic to 80 | *Repeat glucose 205.*  *If the team does not recognize a change in clinical status the embedded participant or RN may state “The blood pressure is “125/90 and the HR is 80”* |
| If participants ask for an ECG |  | *See Appendix D. ECG is provided showing sinus tachycardia without peaked T- waves* |
| If participants ask for a chest x-ray |  | *See Appendix D. Chest x-Xray provided is normal* |
| Patient develops worsening mental status, emesis and tachycardia.  Participants recognize that the patient’s mental status is becoming more altered.  T: 10 minutes | , Patient becomes obtunded, hypertensive to 125/90, and bradycardic to 80. | If asked for, repeat glucose 200  *A: patent*  *B: tachypneic with deep diffuse retractions but lungs are clear to auscultation bilaterally*  *C: bradycardic, 1+ distal pulses, CR ~3 seconds*  *D: GCS 9, eyes closed, withdraws to painful stimuli, irritable*  *E: skin is sweaty and cool*  *GEN-irritable*  *HEENT-pupils 4 mm and sluggishly reactive, NCAT, TMs clear, dry mucous membranes*  *CV-bradycardic to 80, no murmurs, cap refill 3 seconds, 2+ pulses, BP: 125/90*  *LUNG-CTAB, tachypneic, deep respirations*  *AB-soft, nontender, nondistended*  *NEURO- GCS of 9 (E2, V3, M4) eyes open to pain, cries in response to pain, withdraws extremities symmetrically to pain*  *SKIN-sweaty, cool,, no rashes or lesions* |
| *Participants recognize the concern for cerebral edema and initiate cerebral protection measures.* | *If hypertonic saline or mannitol bolus is started, decrease BP to 110/70 and increase HR to 95.* | *Appropriate measures include: elevation of the head of bed, administration of hypertonic saline or mannitol bolus, bag-valve-mask ventilation, preparation for intubate, discussion of head imaging, and consultation of Neurosurgery and Pediatric Critical Care.* |
| *Case completion and handoff to PICU. Participants should identify that the patient requires PICU admission and give handoff to the PICU physician, noting the concern for cerebral edema related to DKA.*  *T: 25 minutes* |  | *Facilitator/embedded participant can play the role of the PICU physician.*  *Example handoff:*  *“This is a 14-month old male presenting with likely DKA in the setting of new onset diabetes, with worsening mental status/agitation concerning for cerebral edema. We have initiated rehydration with 20 mL/kg of NS and an insulin infusion for DKA, as well as cerebral protection measures, consulted neurosurgery, and discussed ordering a head CT. We would like to admit him to the PICU for further treatment.”* |

**Ideal Scenario Flow**

*The participants enter the room to find an irritable 14-month old male in respiratory distress. They immediately place the patient on bedside monitors and recognize that the patient is dehydrated and in respiratory distress. Supplemental O2 is provided, vascular access is obtained, and an IVF bolus is ordered and initiated. After completing a physical examination and obtaining an appropriate history, participants note that the patient’s respiratory status has not improved and labs are suggestive of DKA and likely new onset type 1 diabetes mellitus. The learners reassess the initial 20ml/kg normal saline fluid bolus and provide appropriate slower fluid rehydration and insulin infusion. As these interventions are occurring, the participants recognize the possibility of cerebral edema in the setting of DKA as the patient becomes more agitated and tachycardic with increasing emesis and initiate cerebral protection measures. The participants admit the patient to the PICU with neurosurgical consultation.*

**Anticipated Management Mistakes**

1. *Failure to recognize DKA in a pediatric patient: It can be difficult to diagnose DKA in a toddler with no previous diagnosis of diabetes. If participants do not recognize the possibility of DKA, a facilitator should act as an embedded participant and call out concern for abnormal lab values.*
2. *Failure to administer resuscitative fluids judiciously: In pediatric DKA, there is an increased risk of cerebral edema if the patient rapidly receives more than 40ml/kg in fluid resuscitation or more than 20ml/kg/hr. If fluids are being administered too quickly, the patient should decompensate more quickly with worsening agitation, altered mental status, and increasing emesis. In addition, a facilitator can act as an embedded participant and question the rapid fluid administration.*
3. *Failure to correct acidosis or hyperglycemia at an appropriate rate: Correcting hyperglycemia too rapidly (> 100 mg/dL/hr) via a high rate of insulin administration or correcting acidosis with bicarbonate boluses will increase a pediatric patient’s risk of developing cerebral edema. If the participants administer either an insulin or sodium bicarbonate bolus, the patient should develop signs of cerebral edema with worsening agitation, altered mental status, bradycardia and hypertension.*
4. *Failure to recognize and support the patient’s airway, breathing, and circulation. Most learners reassessed airway, breathing and circulation frequently throughout the scenario and in response to interventions. If participants are not performing repeat assessments, the facilitator may act as an embedded participant and call out a vital sign to prompt participants.*
