# Supplementary material for: Pediatric Emergency Medicine Didactics and Simulation (PEMDAS): Pediatric Diabetic Ketoacidosis
Source: MedEdPORTAL. 2021 Feb 17;17:11098. doi: 10.15766/mep_2374-8265.11098 (PMC7901255; doi:10.15766/mep_2374-8265.11098)
Supplement: Supplementary file 1 — Ped DKA Simulation Case.docxPed DKA Environmental Preparation.docxPed DKA Critical Actions.docxPed DKA ECG CXR Labs.docxPed DKA Debriefing Materials.docxPed DKA TeamSTEPPS Glossary.docxPed DKA Slides.pptxPed DKA Evaluation Form.docx [file mep_2374-8265.11098-s001.zip › C. Ped DKA Critical Actions.docx]

**Appendix C: Ped DKA Critical Actions**

Clincal State #1: Presentation

☐Complete primary patient assessment

☐ Complete secondary patient assessment

☐Place patient on monitors

☐Establish two working IV/IO access points

☐Obtain and interpret diagnostic studies:

☐VBG,

☐POC glucose

☐electrolytes

☐urine ketones (or serum ketones if available)

☐Collect focused history

☐Develop a differential diagnosis for irritability and vomiting in a toddler in the setting of labs suggestive of DKA

☐Begin treatment with fluid rehydration

☐Start 20 mL/kg NS bolus and adjusts fluids accordingly

☐ Begin treatment with insulin administration

Clinical State #2: Worsening Agitation and Vomiting

☐Identify worsening altered mental status in the setting of vomiting and dehydration

☐Perform repeat focused neurologic exam

☐Perform a clinical assessment of cerebral edema risk

☐Initiates maneuvers to reduce potentially increased ICP

☐head of bed elevation

☐mannitol or hypertonic saline

☐hyperventilation)

☐Discuss airway support

☐Obtain a neurosurgical consultation

☐Discuss ordering or obtains a stat head CT

Clinical State #3: Improved Hemodynamics and Admission to PICU

☐Transfer patient to the PICU

☐ Provide hand-off to PICU team
