# Supplementary material for: Pediatric Emergency Medicine Didactics and Simulation (PEMDAS): Pediatric Diabetic Ketoacidosis
Source: MedEdPORTAL. 2021 Feb 17;17:11098. doi: 10.15766/mep_2374-8265.11098 (PMC7901255; doi:10.15766/mep_2374-8265.11098)
Supplement: Supplementary file 1 — Ped DKA Simulation Case.docxPed DKA Environmental Preparation.docxPed DKA Critical Actions.docxPed DKA ECG CXR Labs.docxPed DKA Debriefing Materials.docxPed DKA TeamSTEPPS Glossary.docxPed DKA Slides.pptxPed DKA Evaluation Form.docx [file mep_2374-8265.11098-s001.zip › D. Ped DKA ECG CXR Labs.docx]

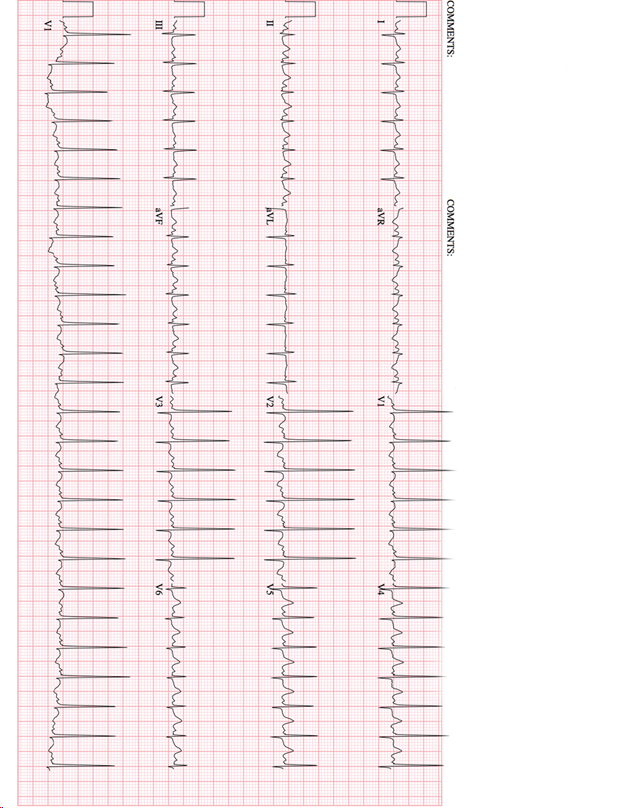
ECG courtesy of Dr. Rebekah Burns, MD


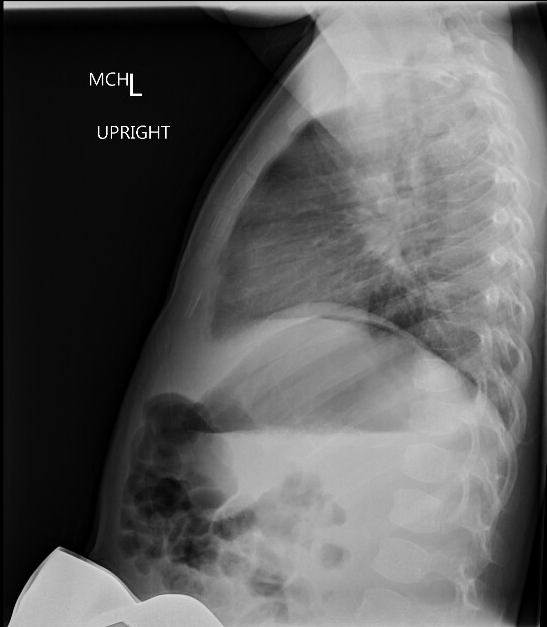


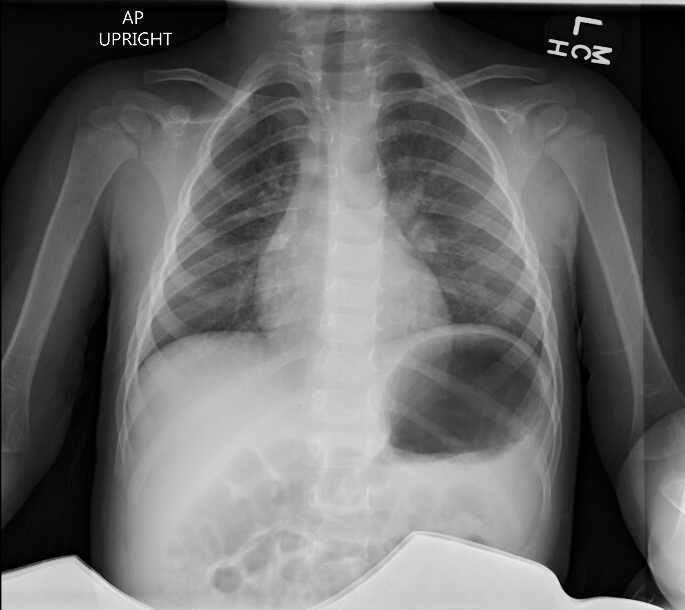


Chest X-ray images courtesy of Dr. Ashley Keilman, MD

Glucose = 451



Venous blood gas

pH 7.11

pCO2 15

pO2 80

bicarbonate 4.9

Base excess -24.7



EPOC Electrolytes:

sodium 132

potassium 5.2

bicarbonate 5

ionized calcium 1.44

lactate 1.8,

Beta-hydroxybutyrate 11.5 mmol/dL

Hematocrit 46

Additional Serum Labs

Magnesium 1.5

Phosphorus 3.2

WBC 18

Hemoglobin 15.7

Hematocrit 47

Platelets 216

BUN 20

Creatinine 0.6

Urinalysis with large ketones, 3+ glucose



Glucose = 390



Glucose = 205
