# Supplementary material for: Pediatric Emergency Medicine Didactics and Simulation (PEMDAS): Pediatric Diabetic Ketoacidosis
Source: MedEdPORTAL. 2021 Feb 17;17:11098. doi: 10.15766/mep_2374-8265.11098 (PMC7901255; doi:10.15766/mep_2374-8265.11098)
Supplement: Supplementary file 1 — Ped DKA Simulation Case.docxPed DKA Environmental Preparation.docxPed DKA Critical Actions.docxPed DKA ECG CXR Labs.docxPed DKA Debriefing Materials.docxPed DKA TeamSTEPPS Glossary.docxPed DKA Slides.pptxPed DKA Evaluation Form.docx [file mep_2374-8265.11098-s001.zip › G. Ped DKA Slides.pptx]

## Slide 1
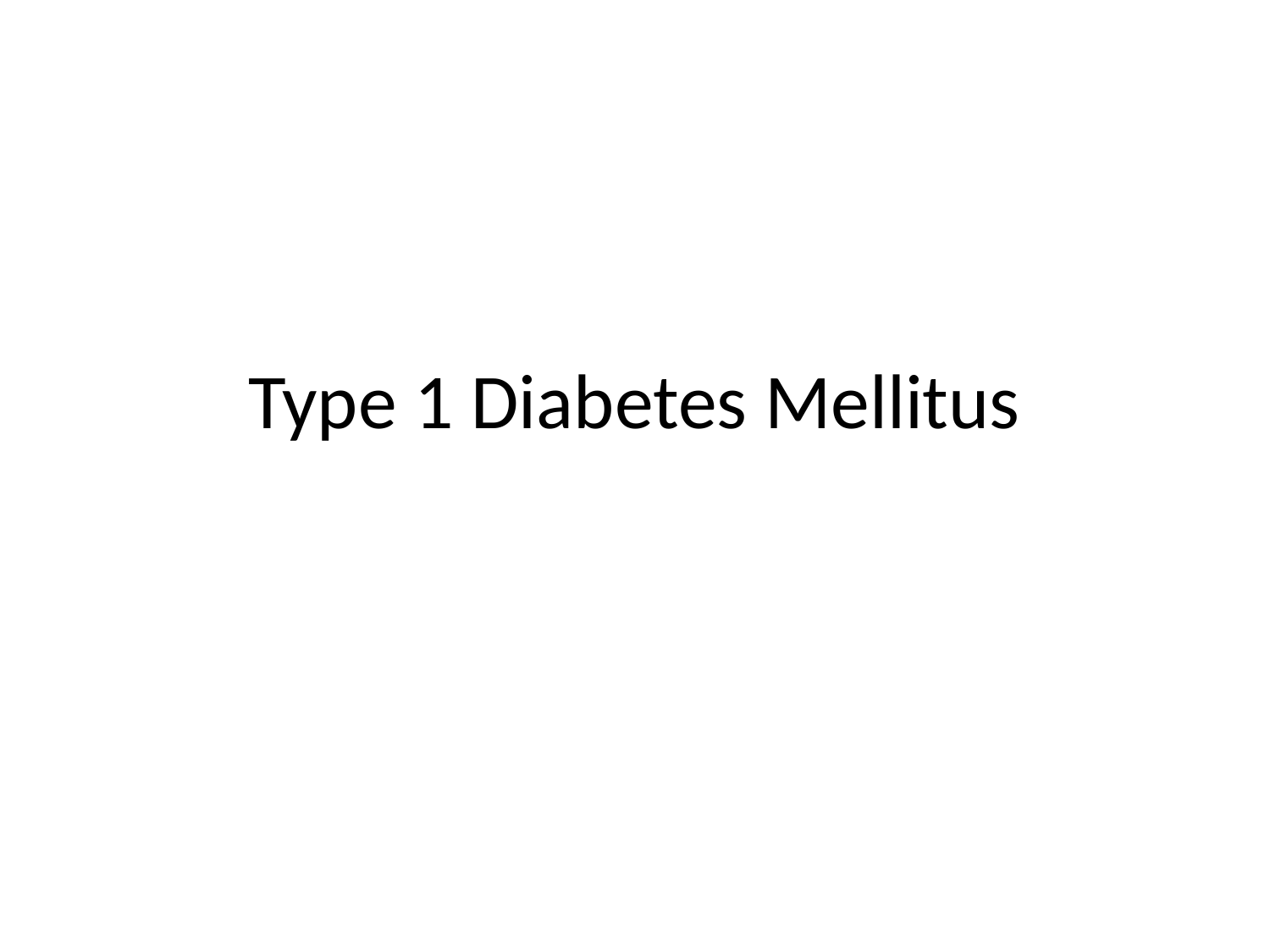

# Type 1 Diabetes Mellitus

## Slide 2
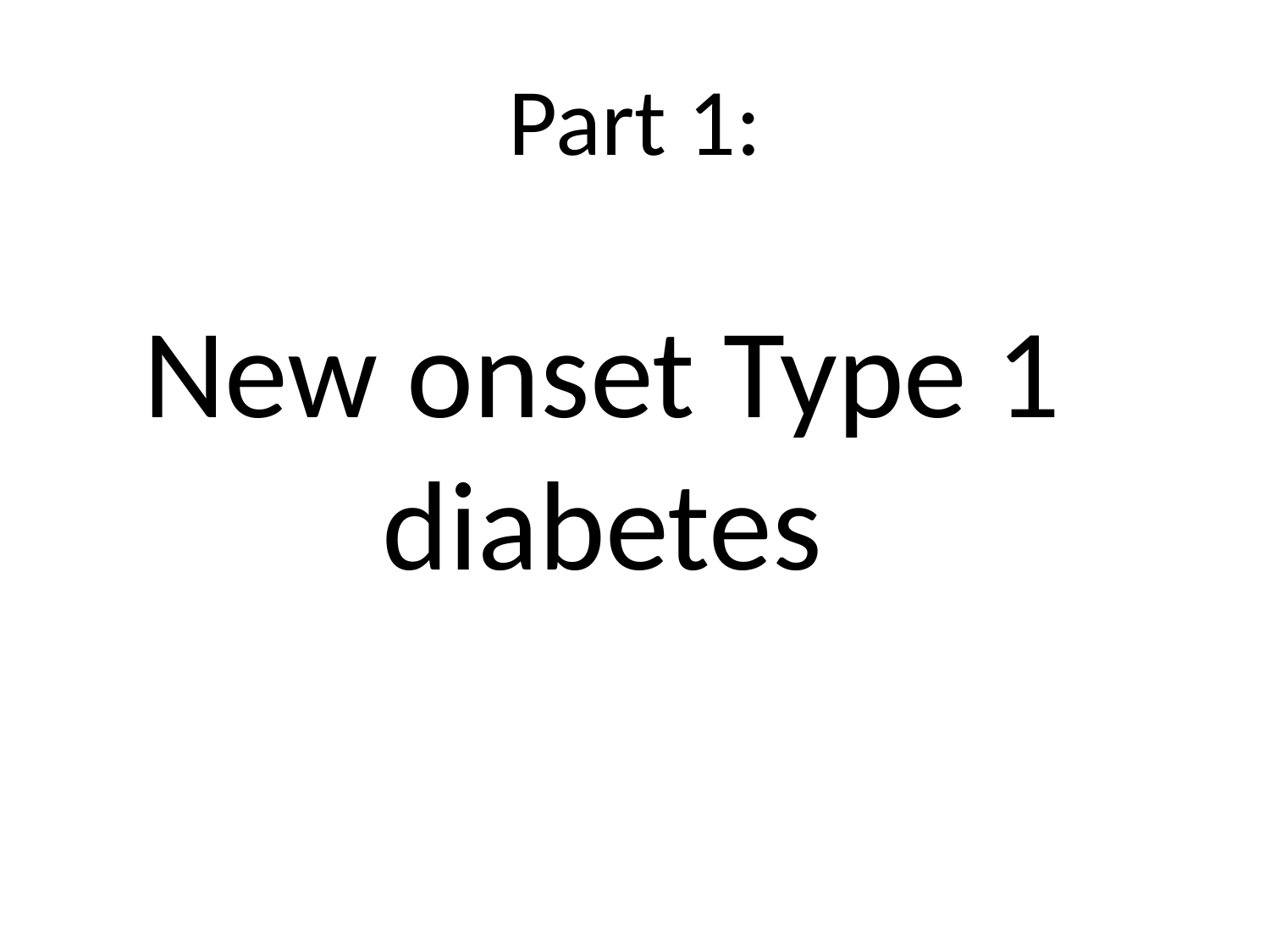

# Part 1:
New onset Type 1 diabetes

## Slide 3
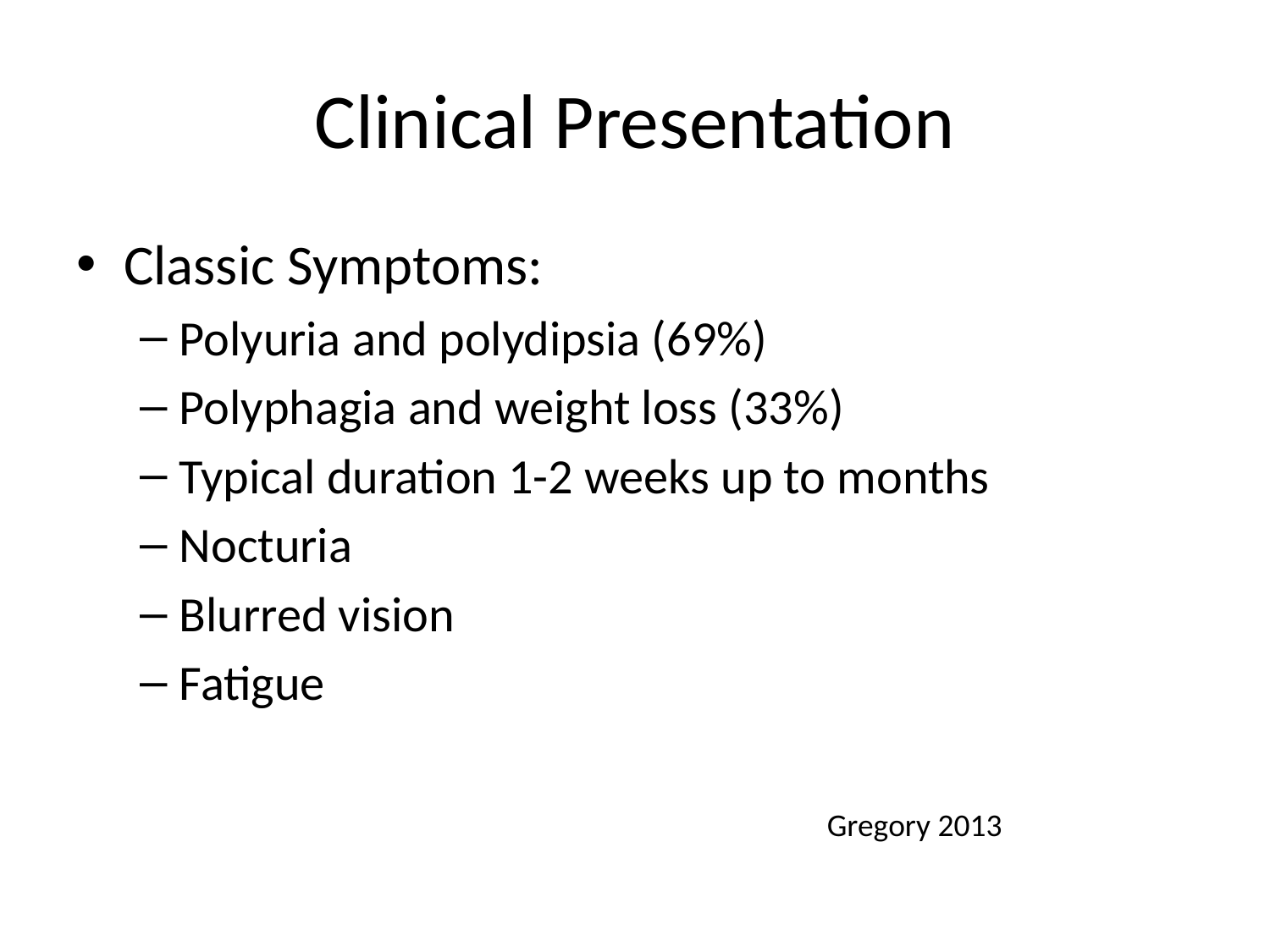

# Clinical Presentation
Classic Symptoms:
Polyuria and polydipsia (69%)
Polyphagia and weight loss (33%)
Typical duration 1-2 weeks up to months
Nocturia
Blurred vision
Fatigue
Gregory 2013

## Slide 4
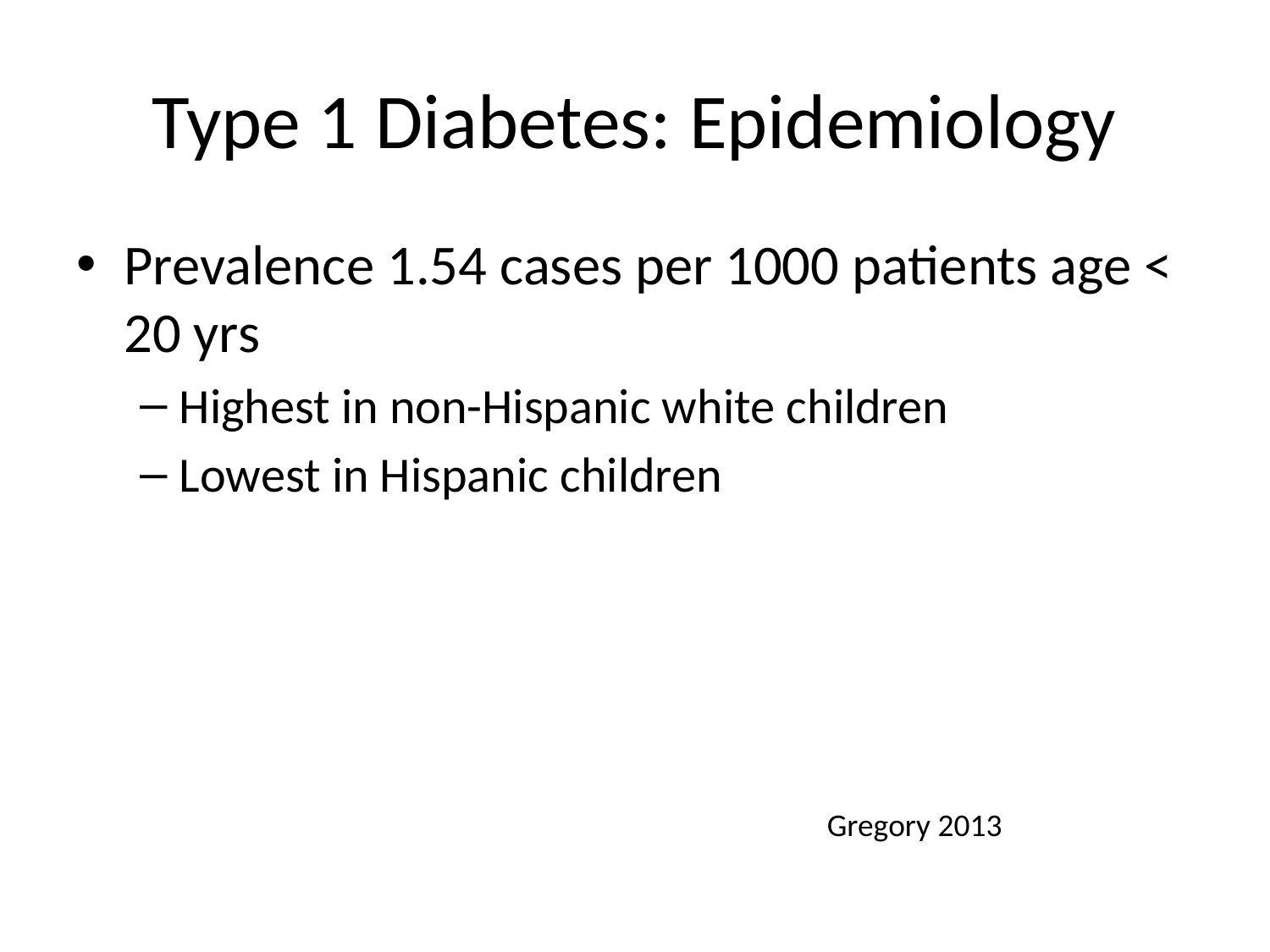

# Type 1 Diabetes: Epidemiology
Prevalence 1.54 cases per 1000 patients age < 20 yrs
Highest in non-Hispanic white children
Lowest in Hispanic children
Gregory 2013

## Slide 5
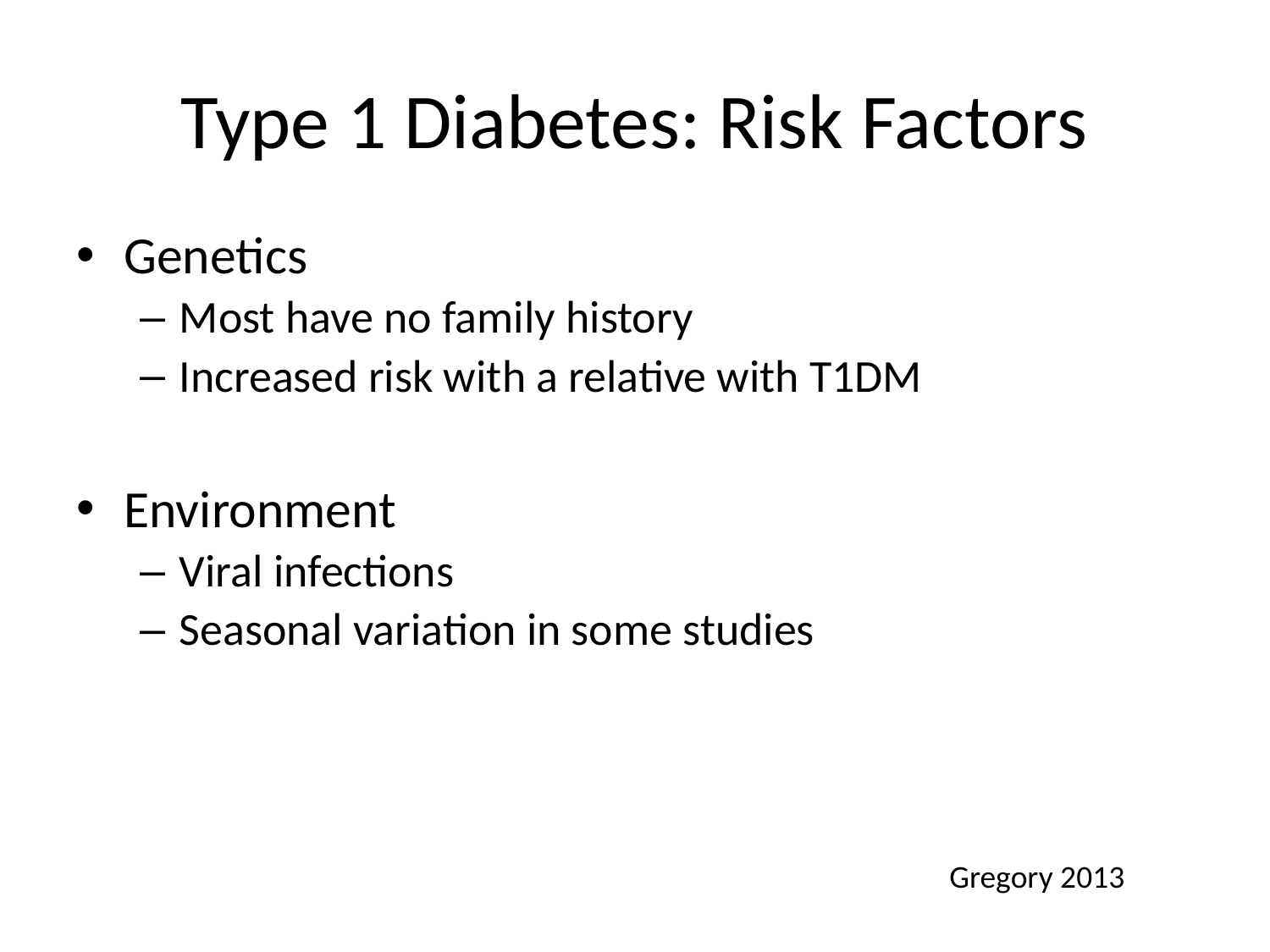

# Type 1 Diabetes: Risk Factors
Genetics
Most have no family history
Increased risk with a relative with T1DM
Environment
Viral infections
Seasonal variation in some studies
Gregory 2013

## Slide 6
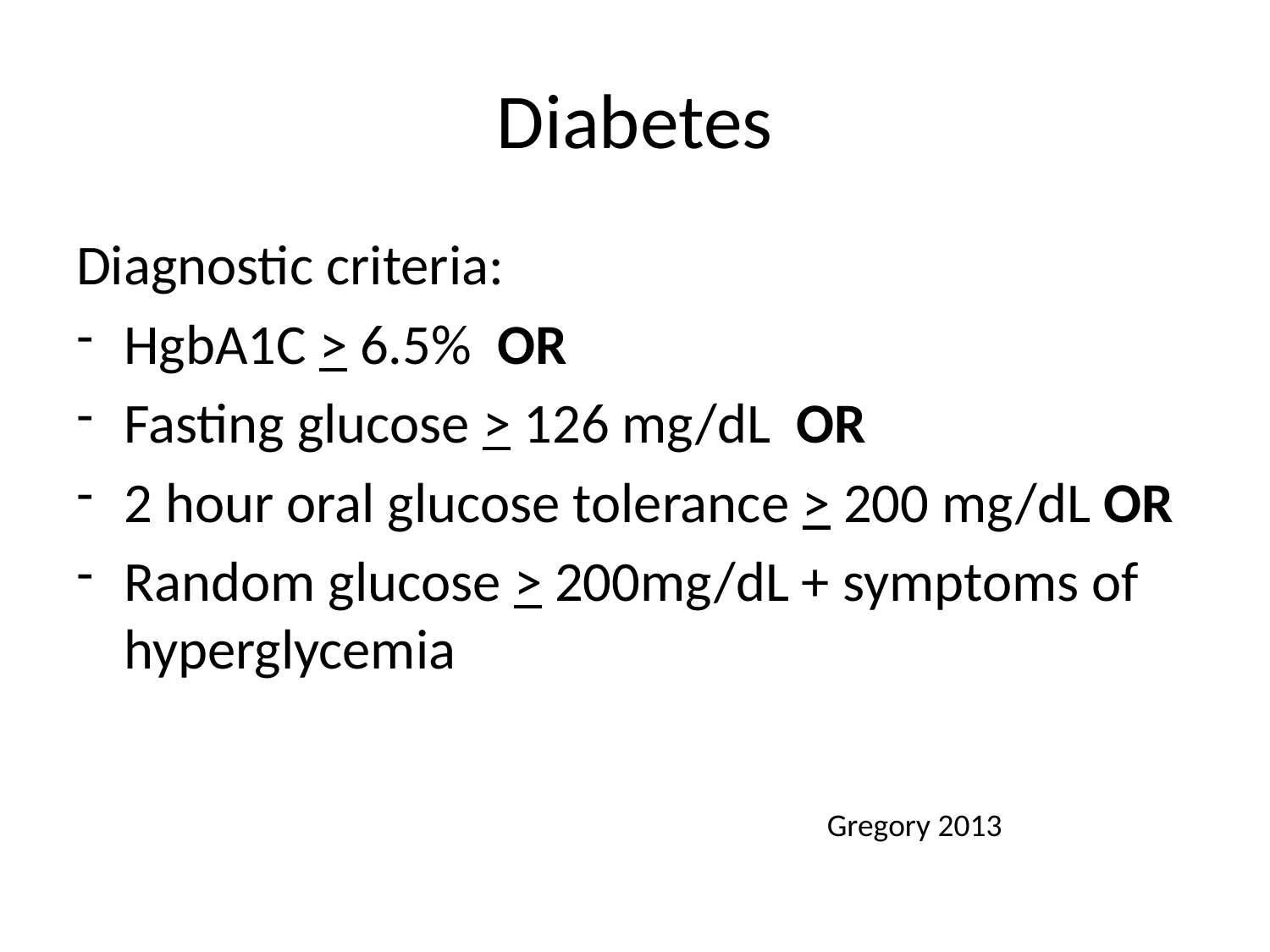

# Diabetes
Diagnostic criteria:
HgbA1C > 6.5% OR
Fasting glucose > 126 mg/dL OR
2 hour oral glucose tolerance > 200 mg/dL OR
Random glucose > 200mg/dL + symptoms of hyperglycemia
Gregory 2013

## Slide 7
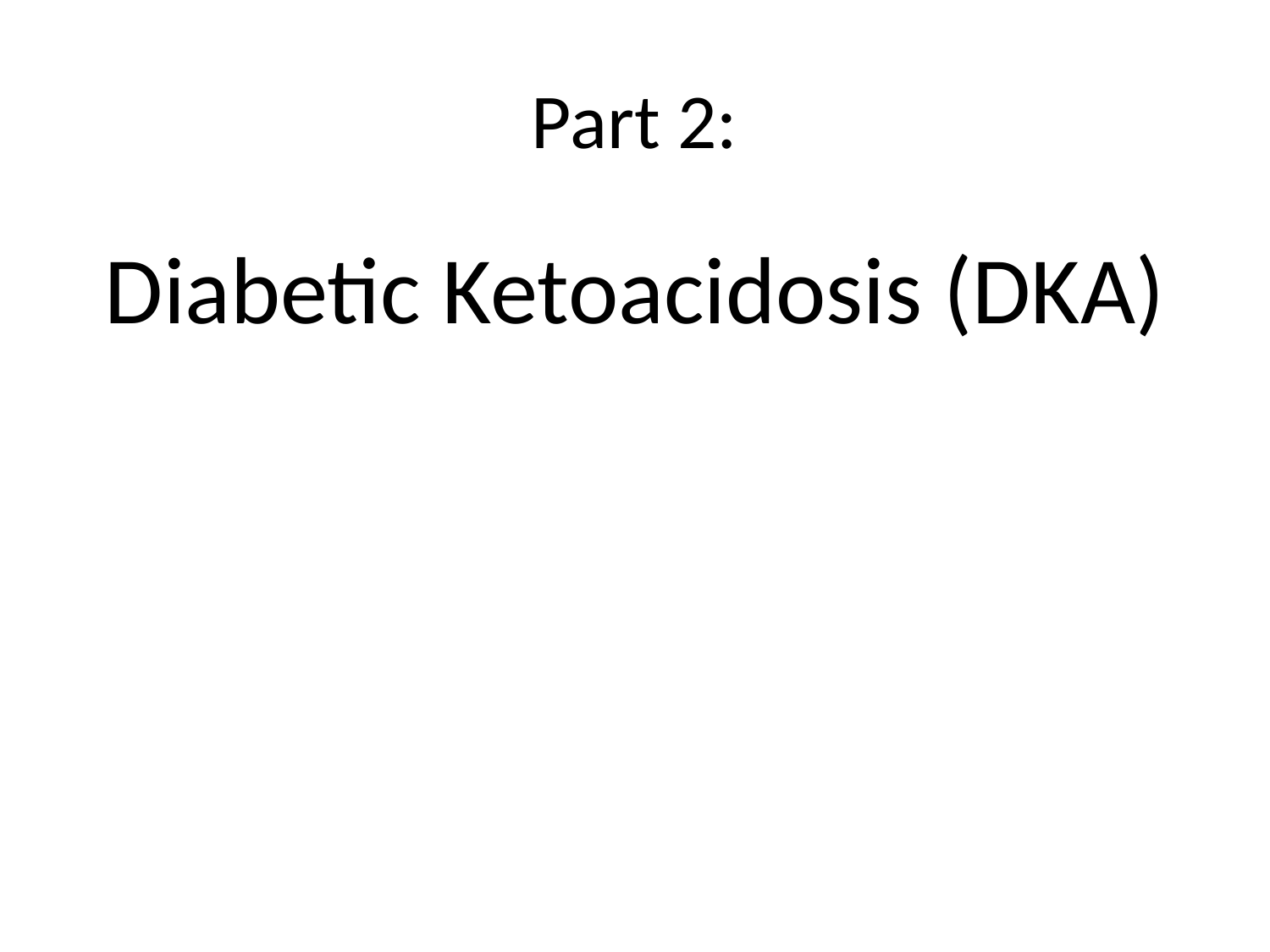

# Part 2:
Diabetic Ketoacidosis (DKA)

## Slide 8
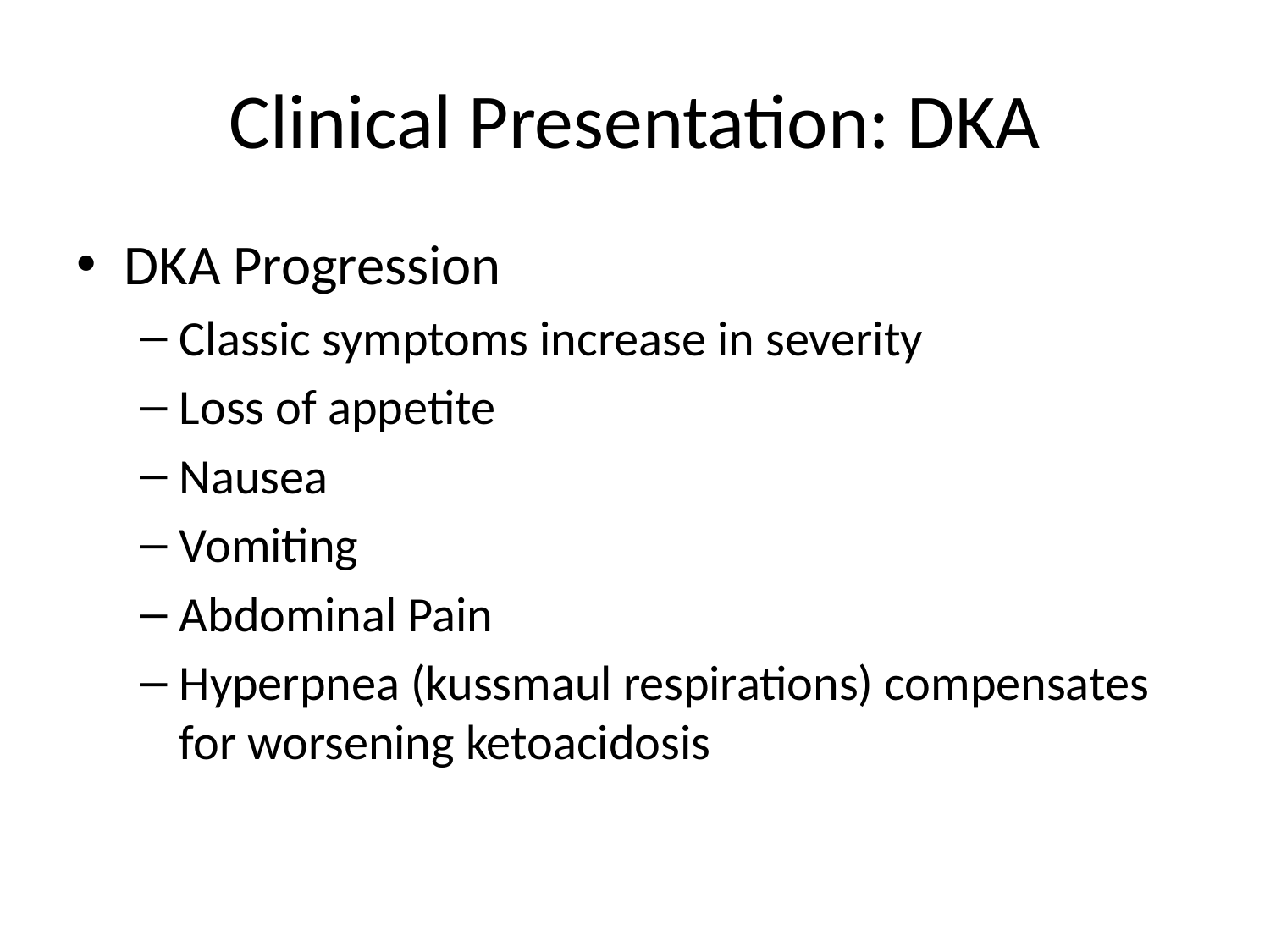

# Clinical Presentation: DKA
DKA Progression
Classic symptoms increase in severity
Loss of appetite
Nausea
Vomiting
Abdominal Pain
Hyperpnea (kussmaul respirations) compensates for worsening ketoacidosis

## Slide 9
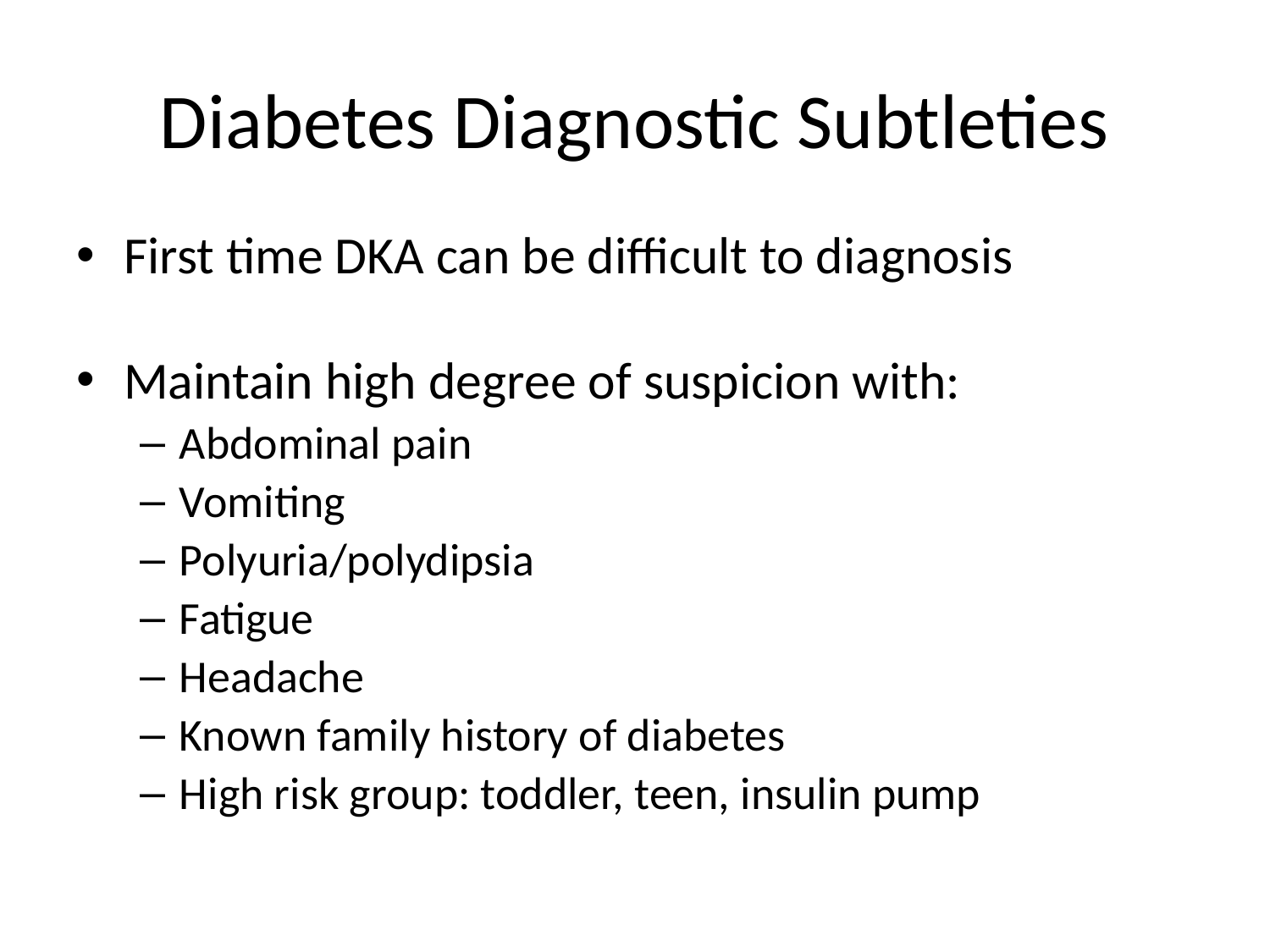

# Diabetes Diagnostic Subtleties
First time DKA can be difficult to diagnosis
Maintain high degree of suspicion with:
Abdominal pain
Vomiting
Polyuria/polydipsia
Fatigue
Headache
Known family history of diabetes
High risk group: toddler, teen, insulin pump

## Slide 10
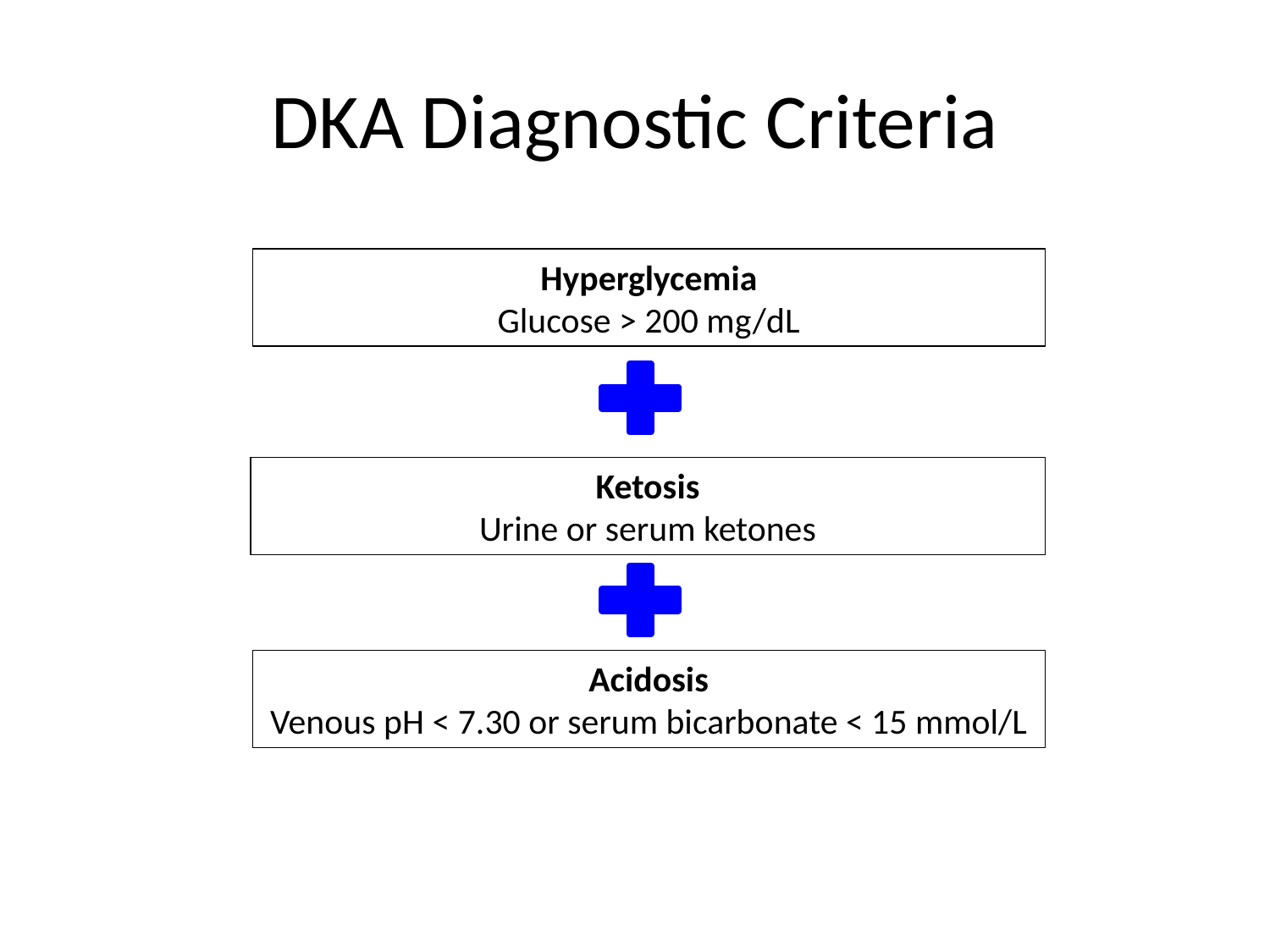

# DKA Diagnostic Criteria
Hyperglycemia
Glucose > 200 mg/dL
Ketosis
Urine or serum ketones
Acidosis
Venous pH < 7.30 or serum bicarbonate < 15 mmol/L

## Slide 11
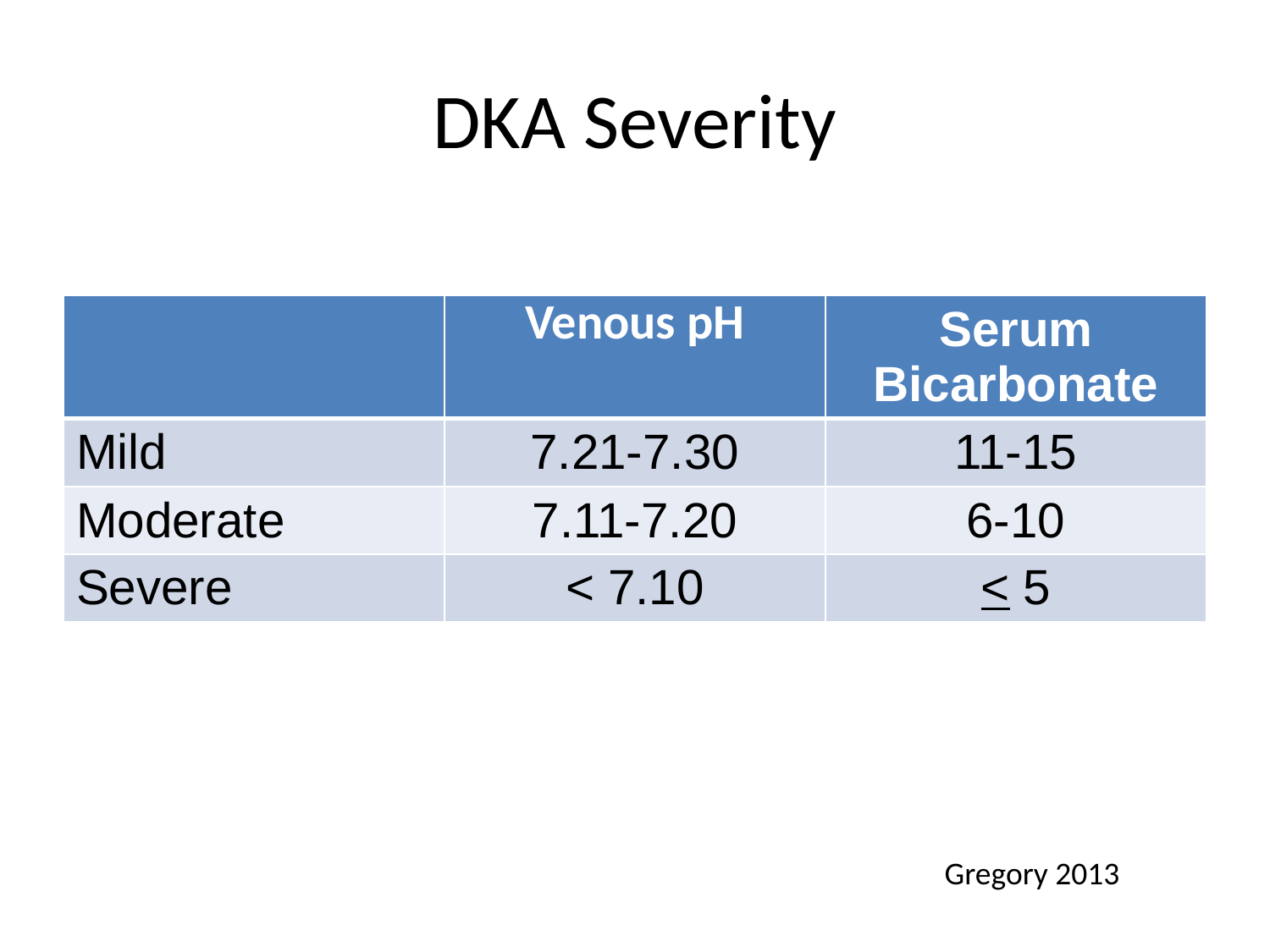

# DKA Severity
| | Venous pH | Serum Bicarbonate |
| --- | --- | --- |
| Mild | 7.21-7.30 | 11-15 |
| Moderate | 7.11-7.20 | 6-10 |
| Severe | < 7.10 | < 5 |
Gregory 2013

## Slide 12
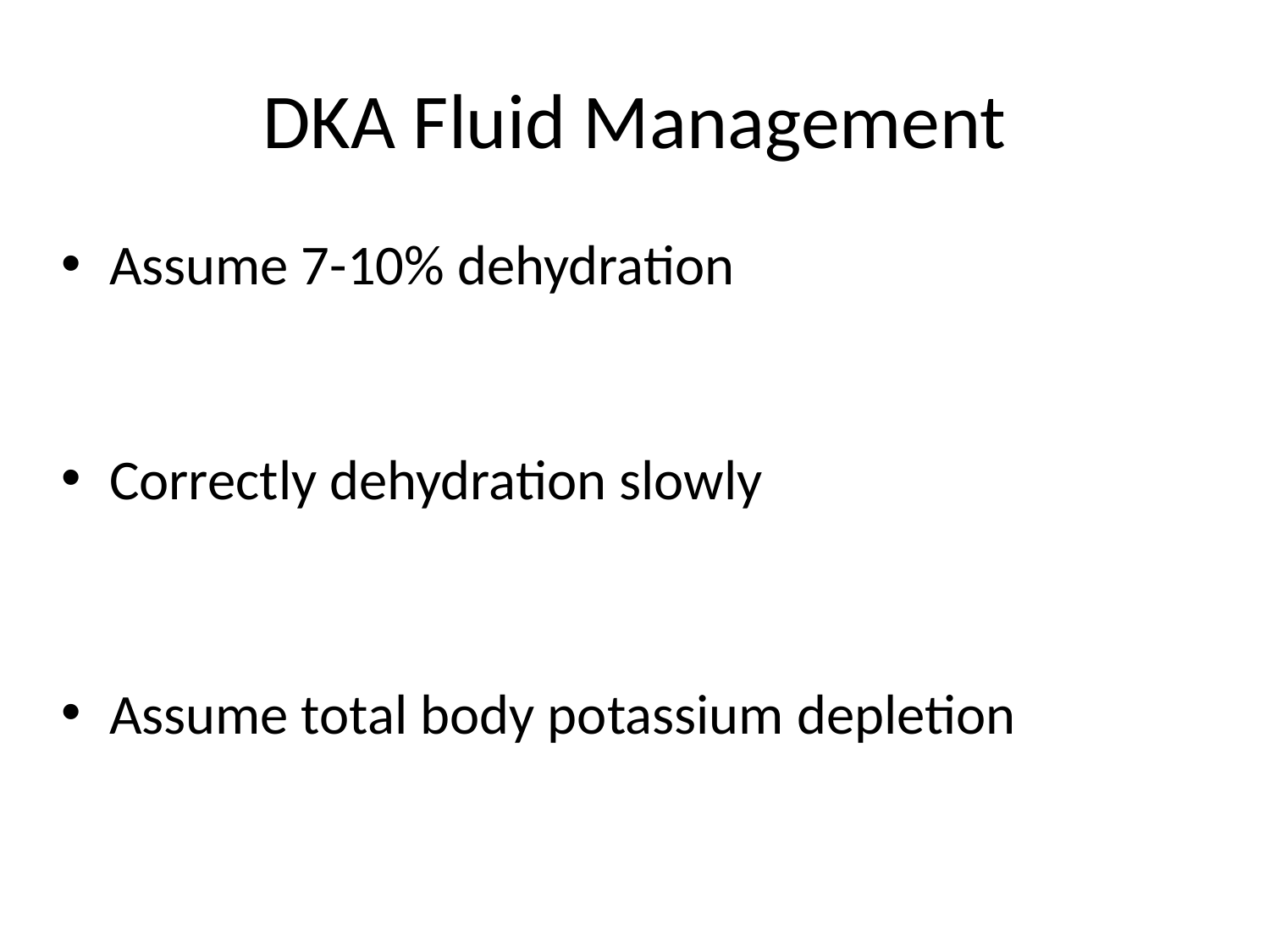

# DKA Fluid Management
Assume 7-10% dehydration
Correctly dehydration slowly
Assume total body potassium depletion

## Slide 13
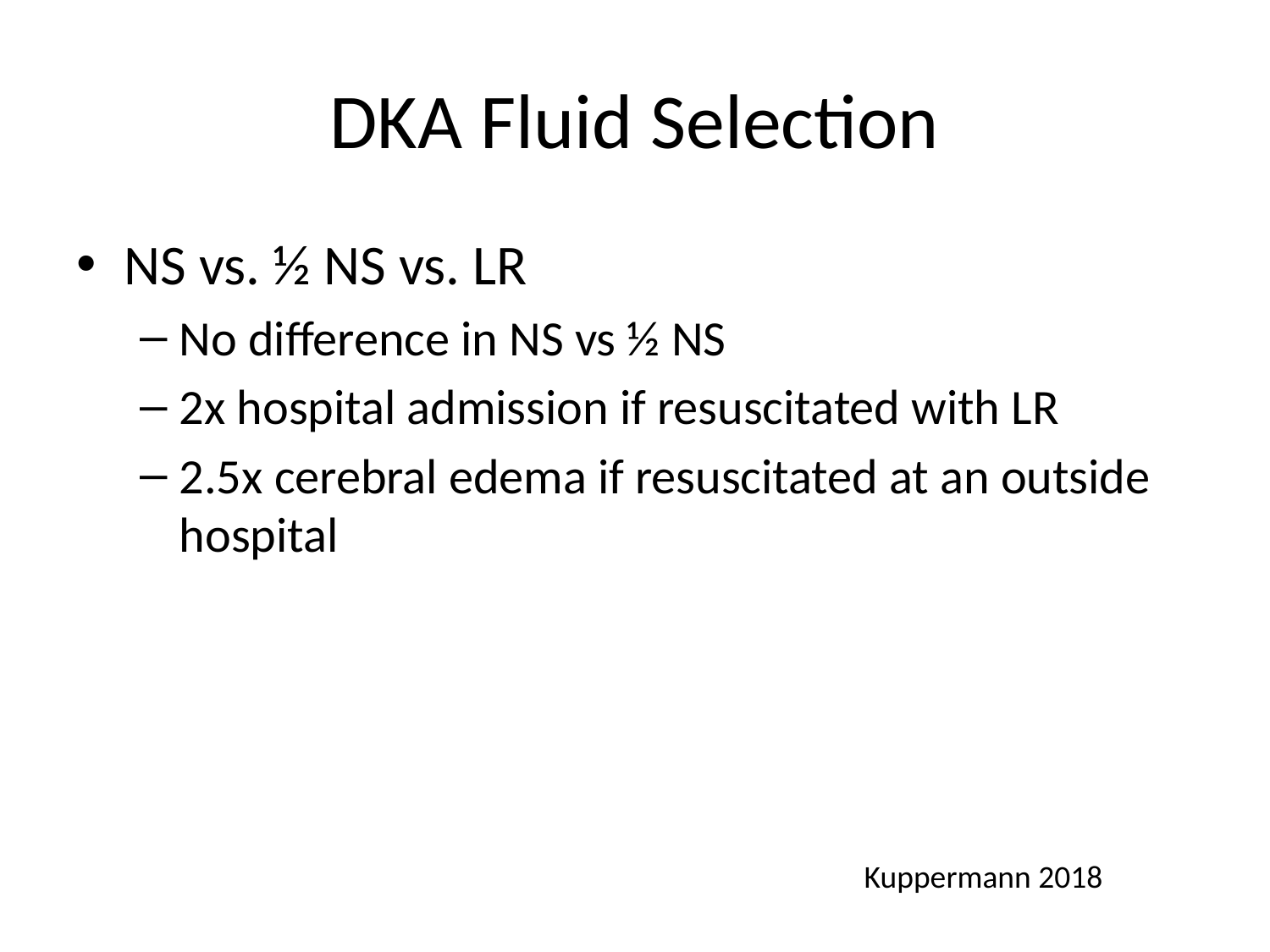

# DKA Fluid Selection
NS vs. ½ NS vs. LR
No difference in NS vs ½ NS
2x hospital admission if resuscitated with LR
2.5x cerebral edema if resuscitated at an outside hospital
Kuppermann 2018

## Slide 14
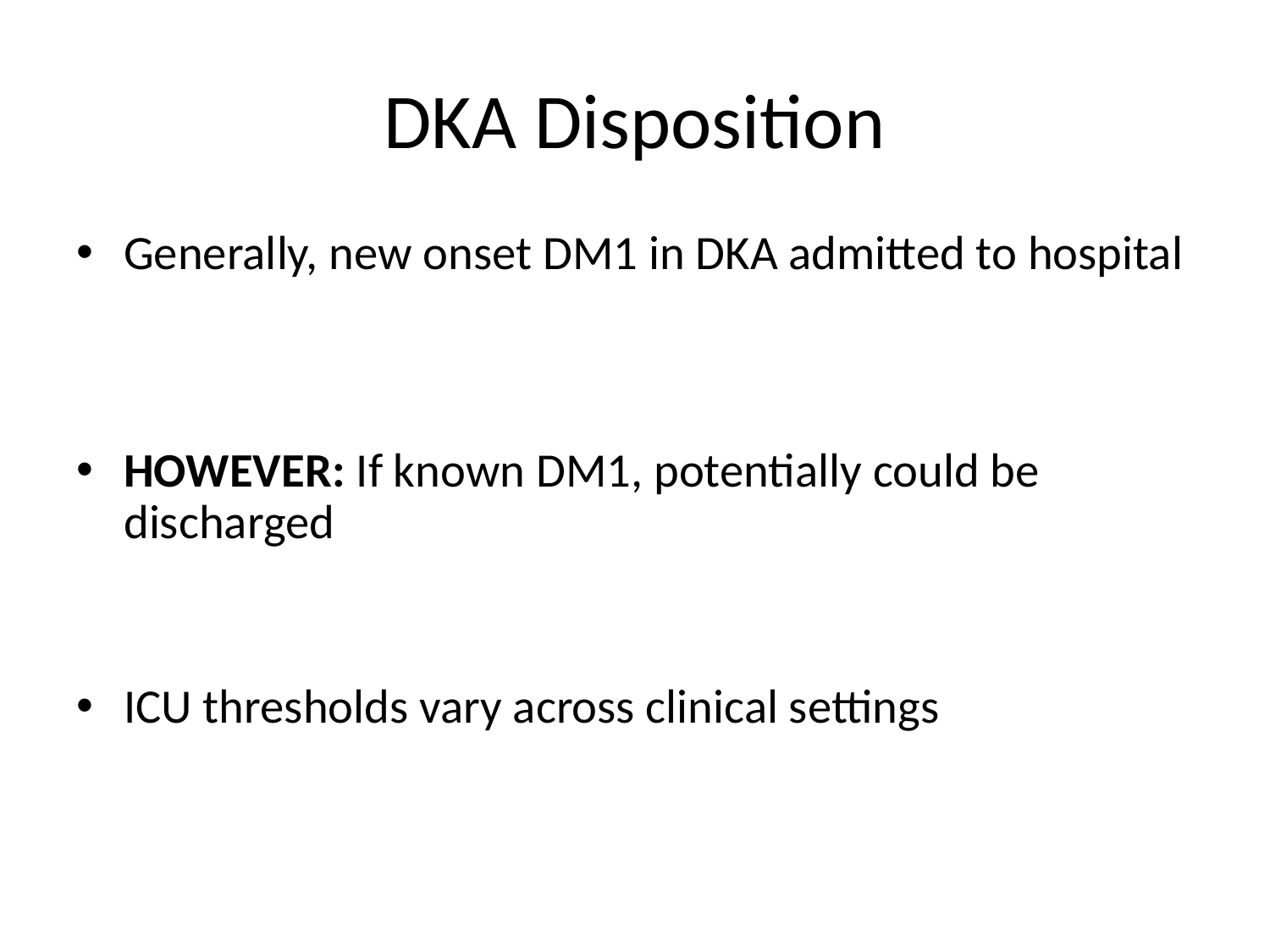

# DKA Disposition
Generally, new onset DM1 in DKA admitted to hospital
HOWEVER: If known DM1, potentially could be discharged
ICU thresholds vary across clinical settings

## Slide 15
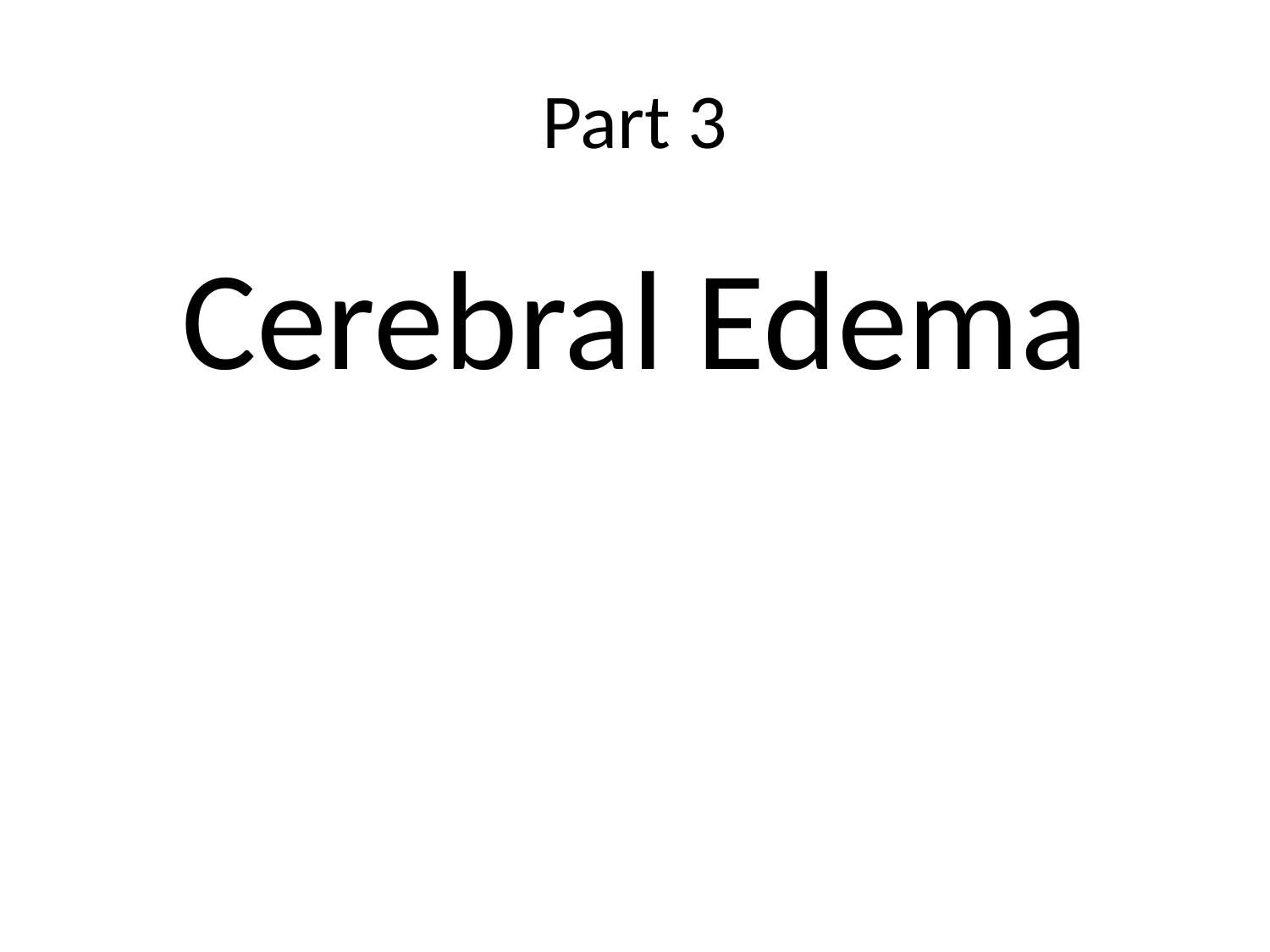

# Part 3
Cerebral Edema

## Slide 16
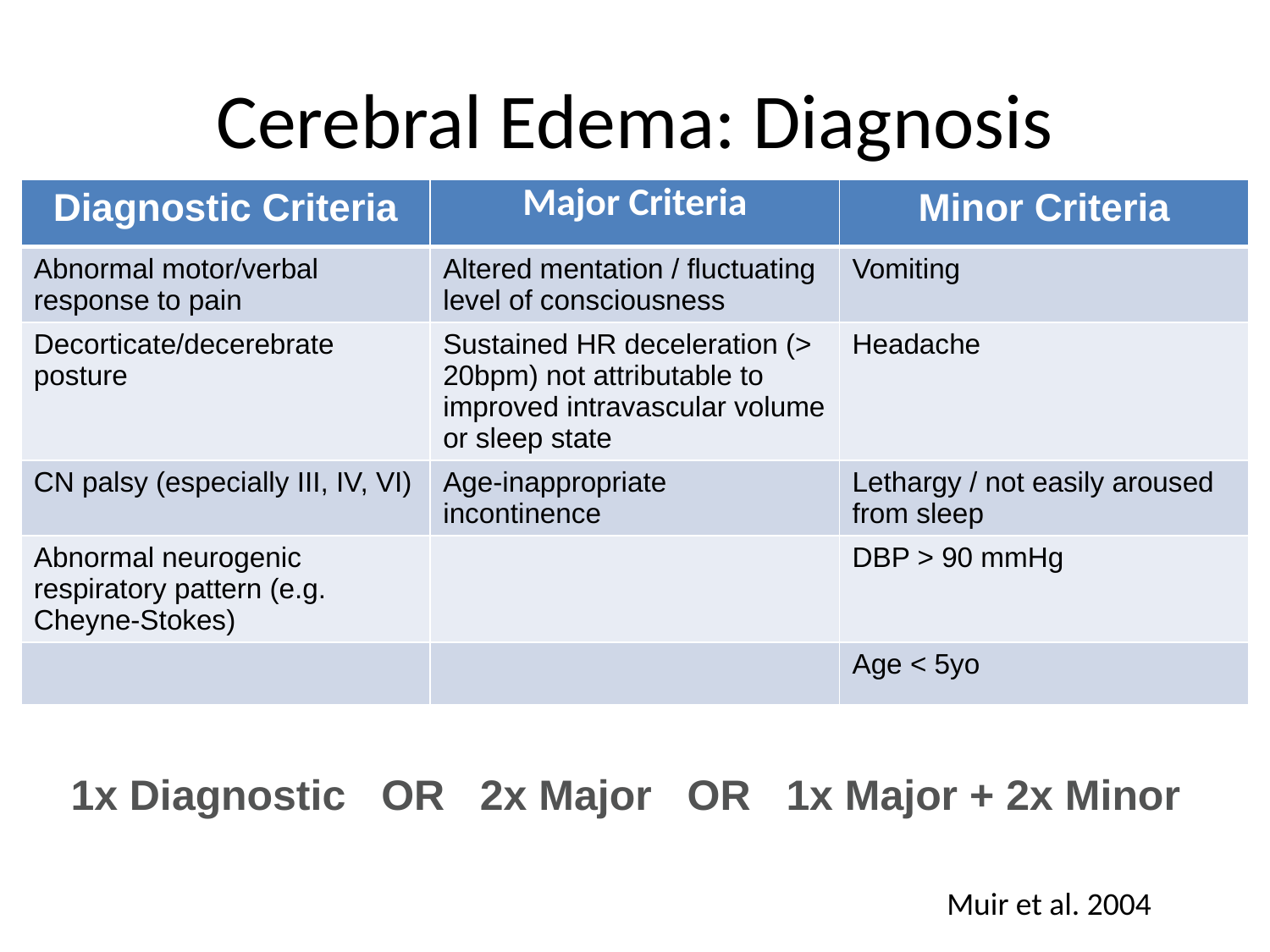

# Cerebral Edema: Diagnosis
| Diagnostic Criteria | Major Criteria | Minor Criteria |
| --- | --- | --- |
| Abnormal motor/verbal response to pain | Altered mentation / fluctuating level of consciousness | Vomiting |
| Decorticate/decerebrate posture | Sustained HR deceleration (> 20bpm) not attributable to improved intravascular volume or sleep state | Headache |
| CN palsy (especially III, IV, VI) | Age-inappropriate incontinence | Lethargy / not easily aroused from sleep |
| Abnormal neurogenic respiratory pattern (e.g. Cheyne-Stokes) | | DBP > 90 mmHg |
| | | Age < 5yo |
1x Diagnostic OR 2x Major OR 1x Major + 2x Minor
Muir et al. 2004

## Slide 17
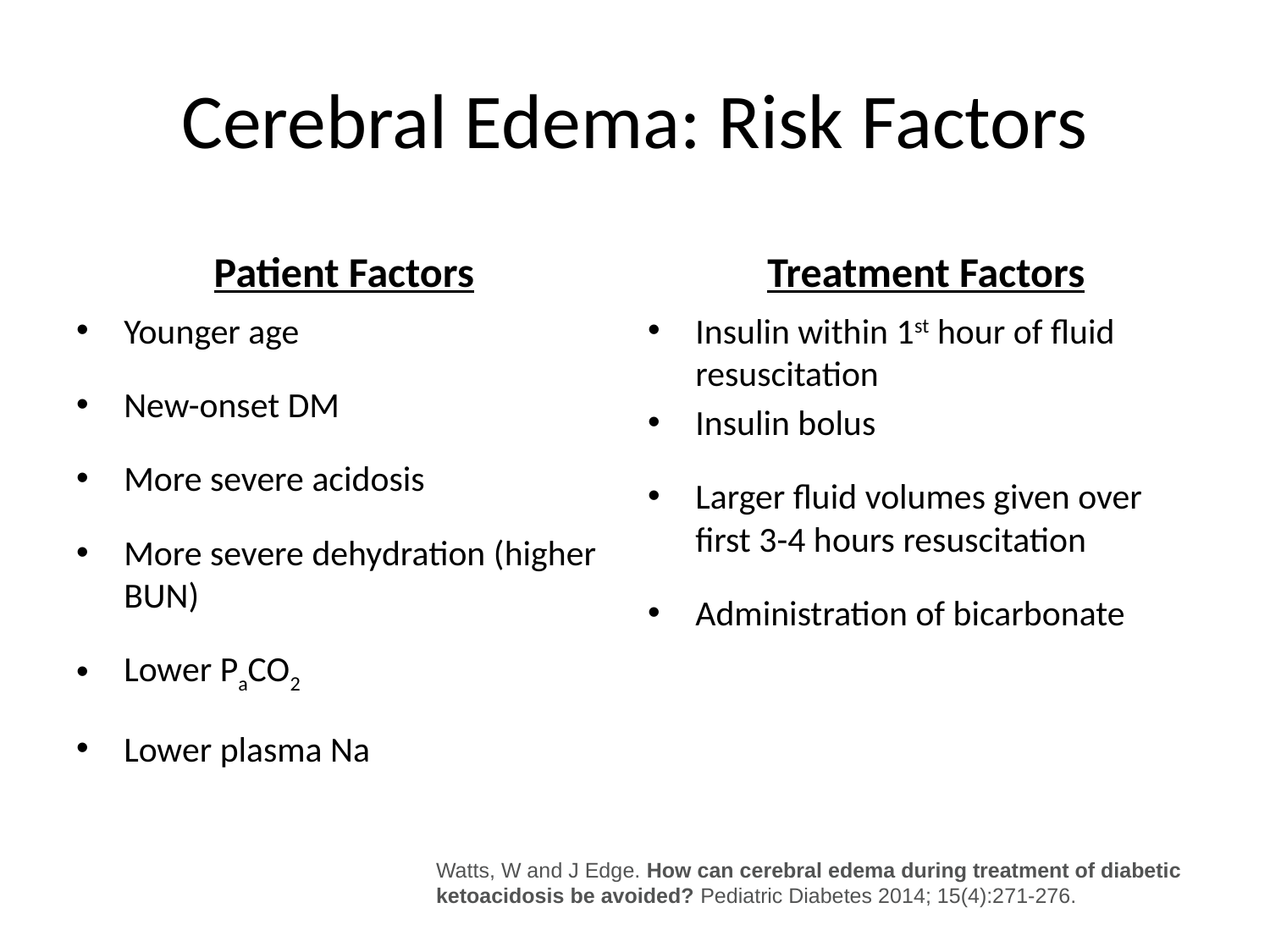

# Cerebral Edema: Risk Factors
Patient Factors
Treatment Factors
Younger age
New-onset DM
More severe acidosis
More severe dehydration (higher BUN)
Lower PaCO2
Lower plasma Na
Insulin within 1st hour of fluid resuscitation
Insulin bolus
Larger fluid volumes given over first 3-4 hours resuscitation
Administration of bicarbonate
Watts, W and J Edge. How can cerebral edema during treatment of diabetic ketoacidosis be avoided? Pediatric Diabetes 2014; 15(4):271-276.

## Slide 18
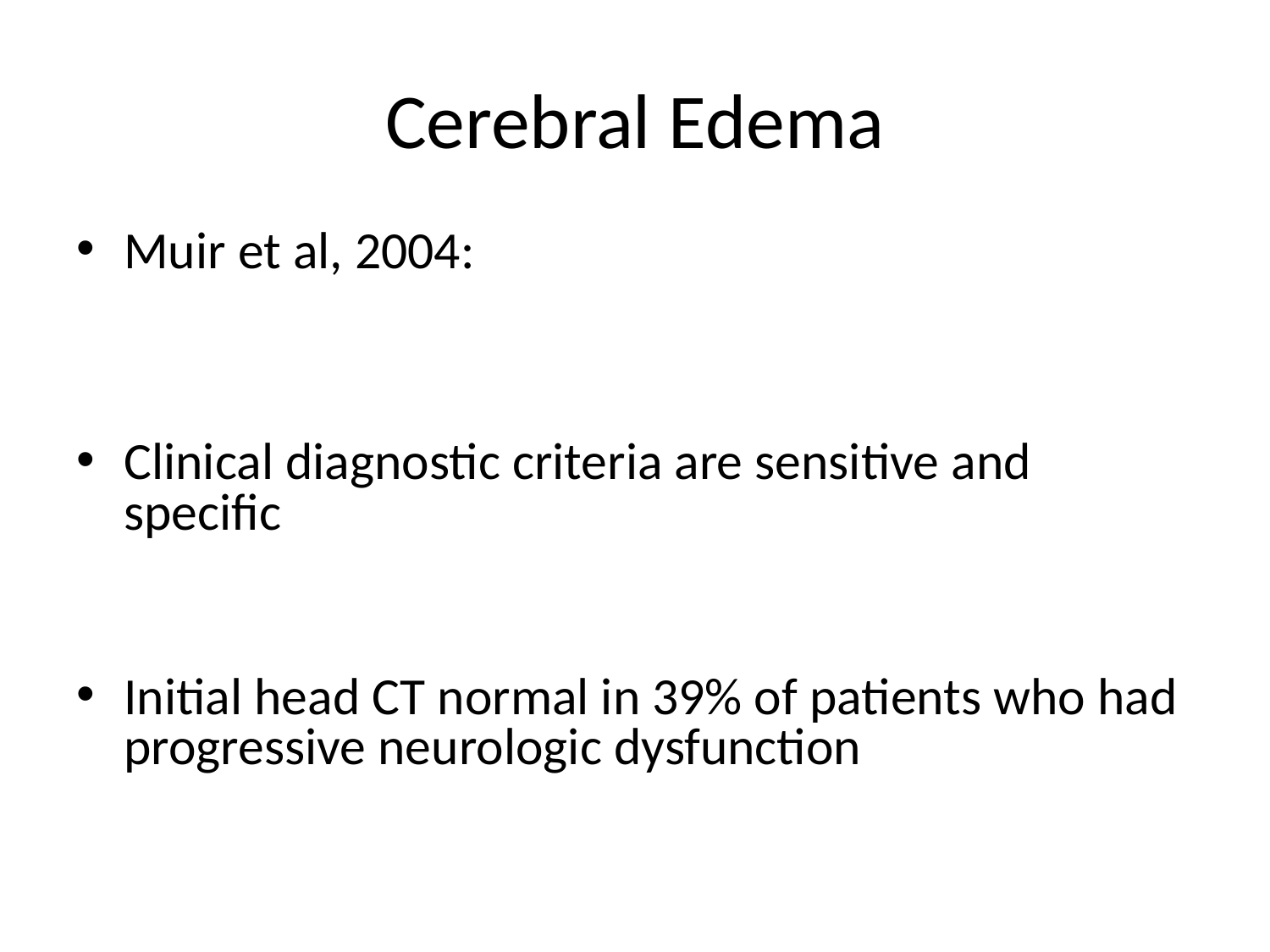

# Cerebral Edema
Muir et al, 2004:
Clinical diagnostic criteria are sensitive and specific
Initial head CT normal in 39% of patients who had progressive neurologic dysfunction

## Slide 19
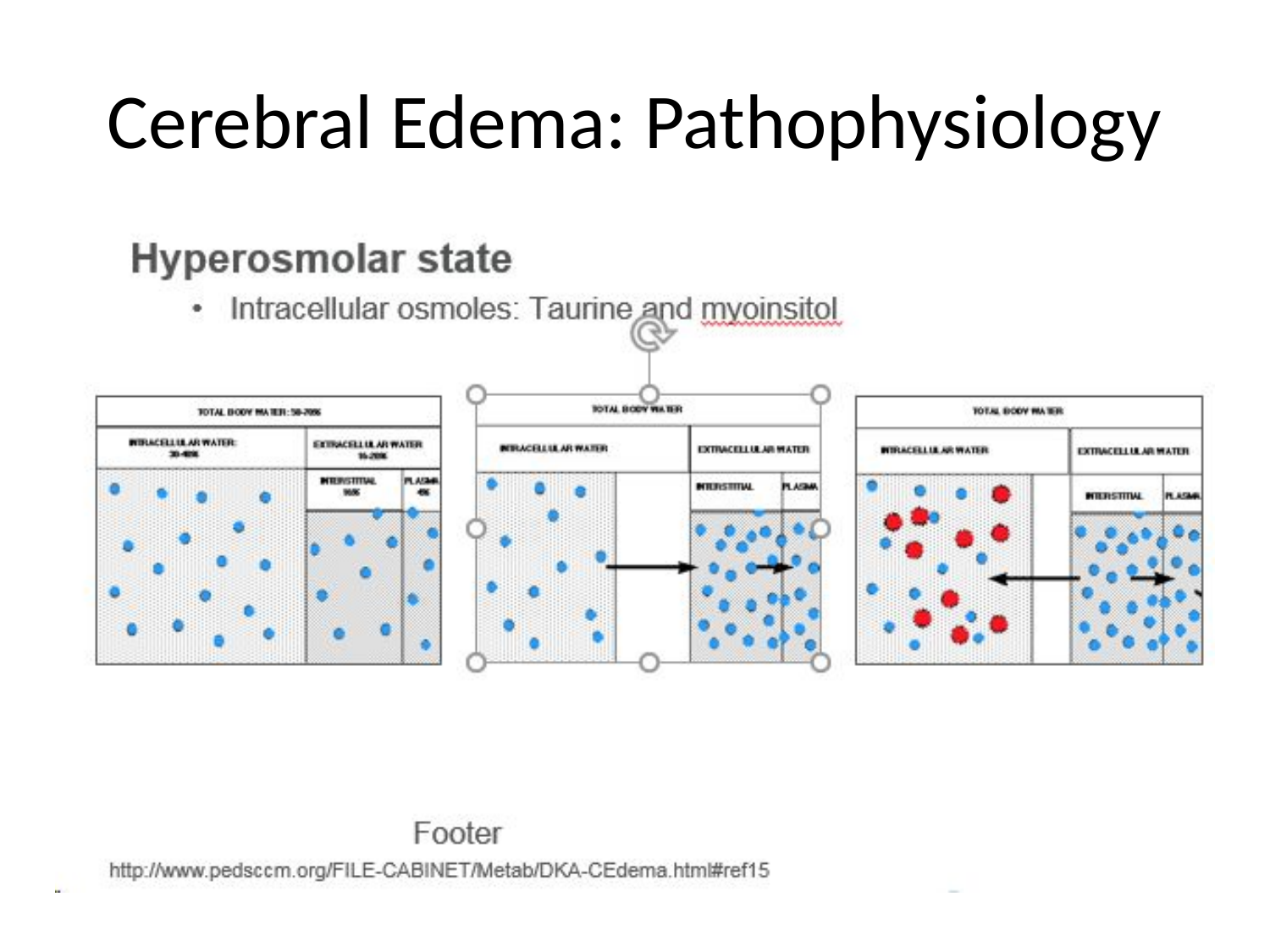

# Cerebral Edema: Pathophysiology

## Slide 20
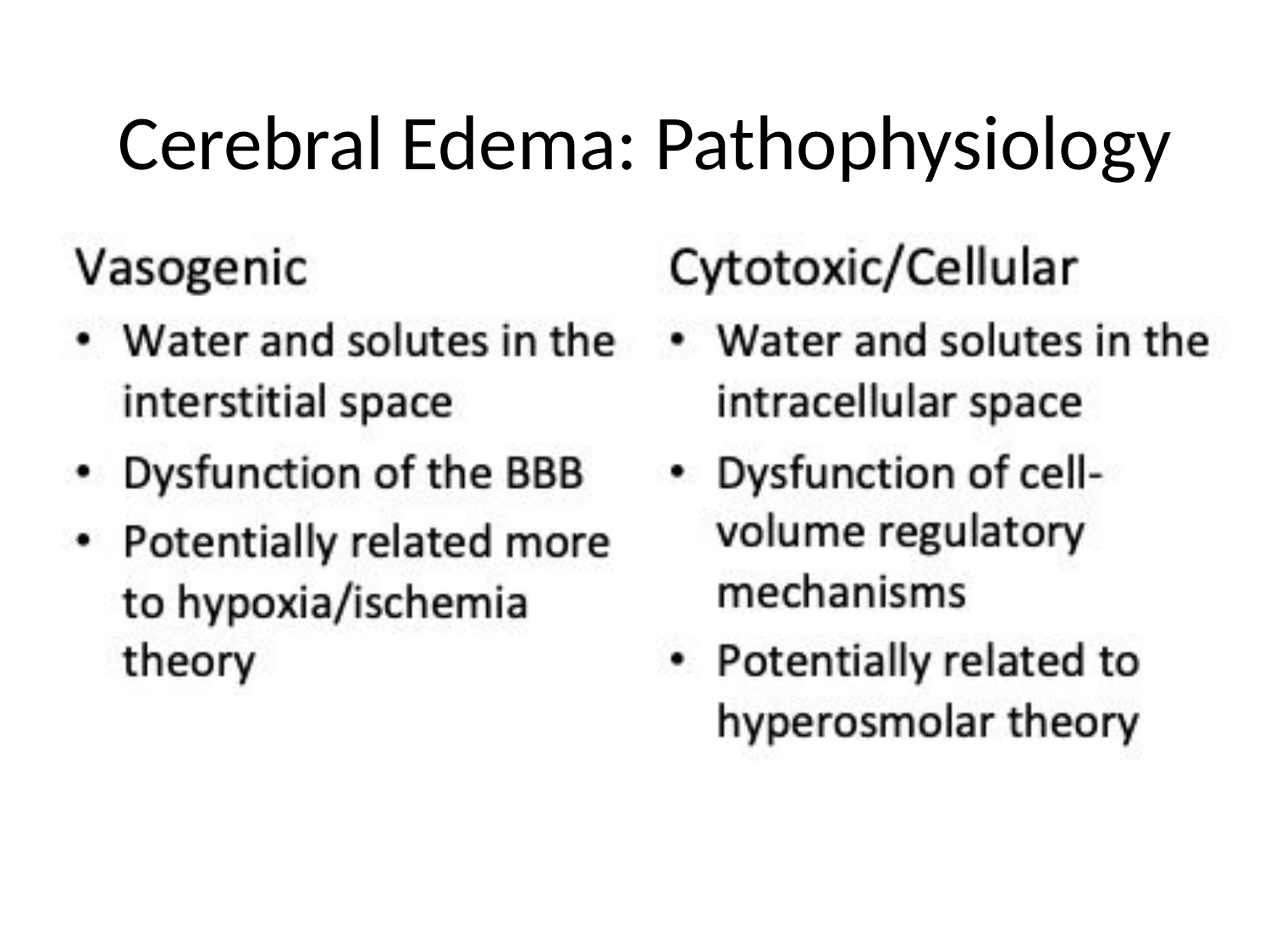

Cerebral Edema: Pathophysiology

## Slide 21
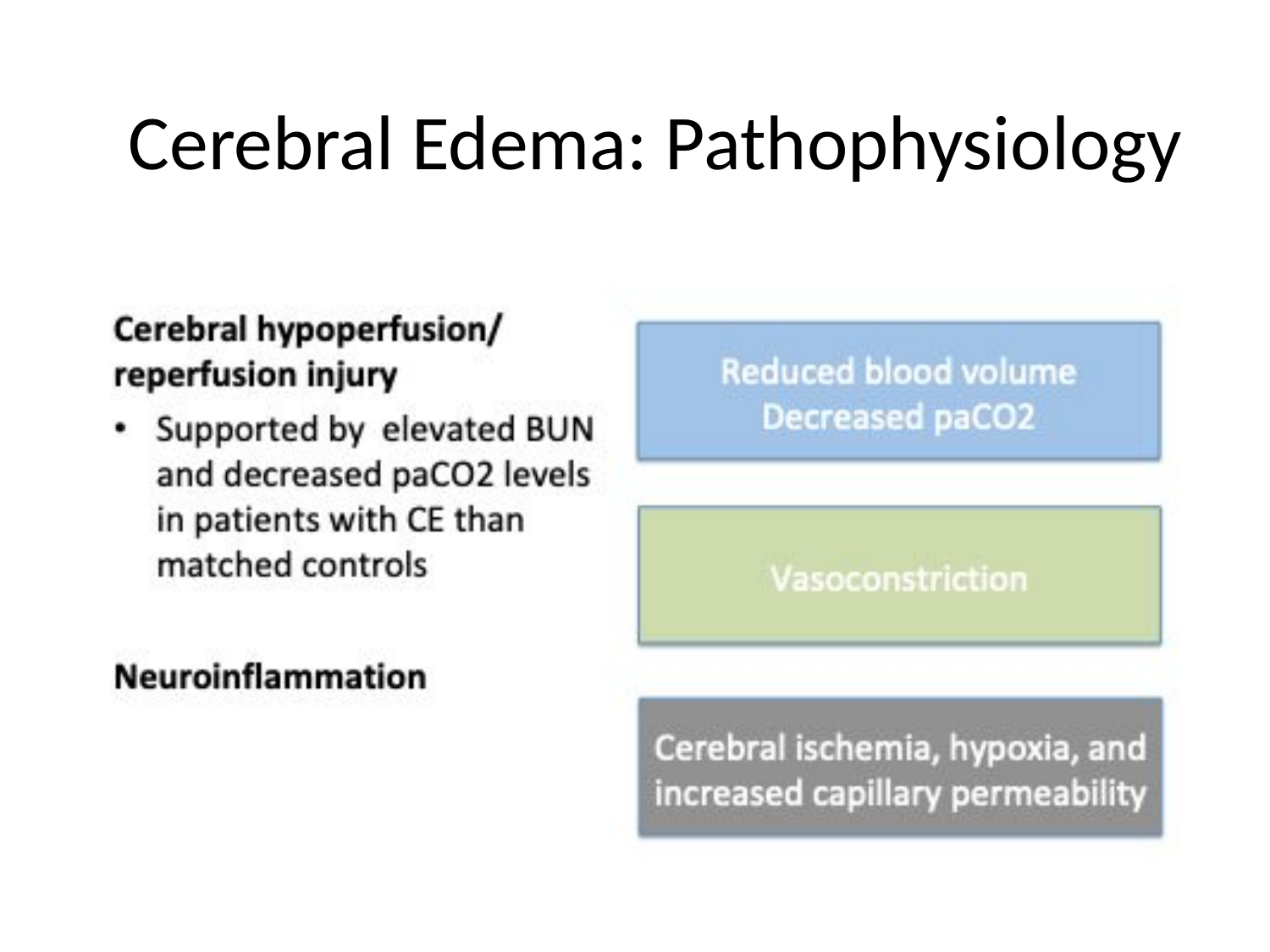

Cerebral Edema: Pathophysiology

## Slide 22
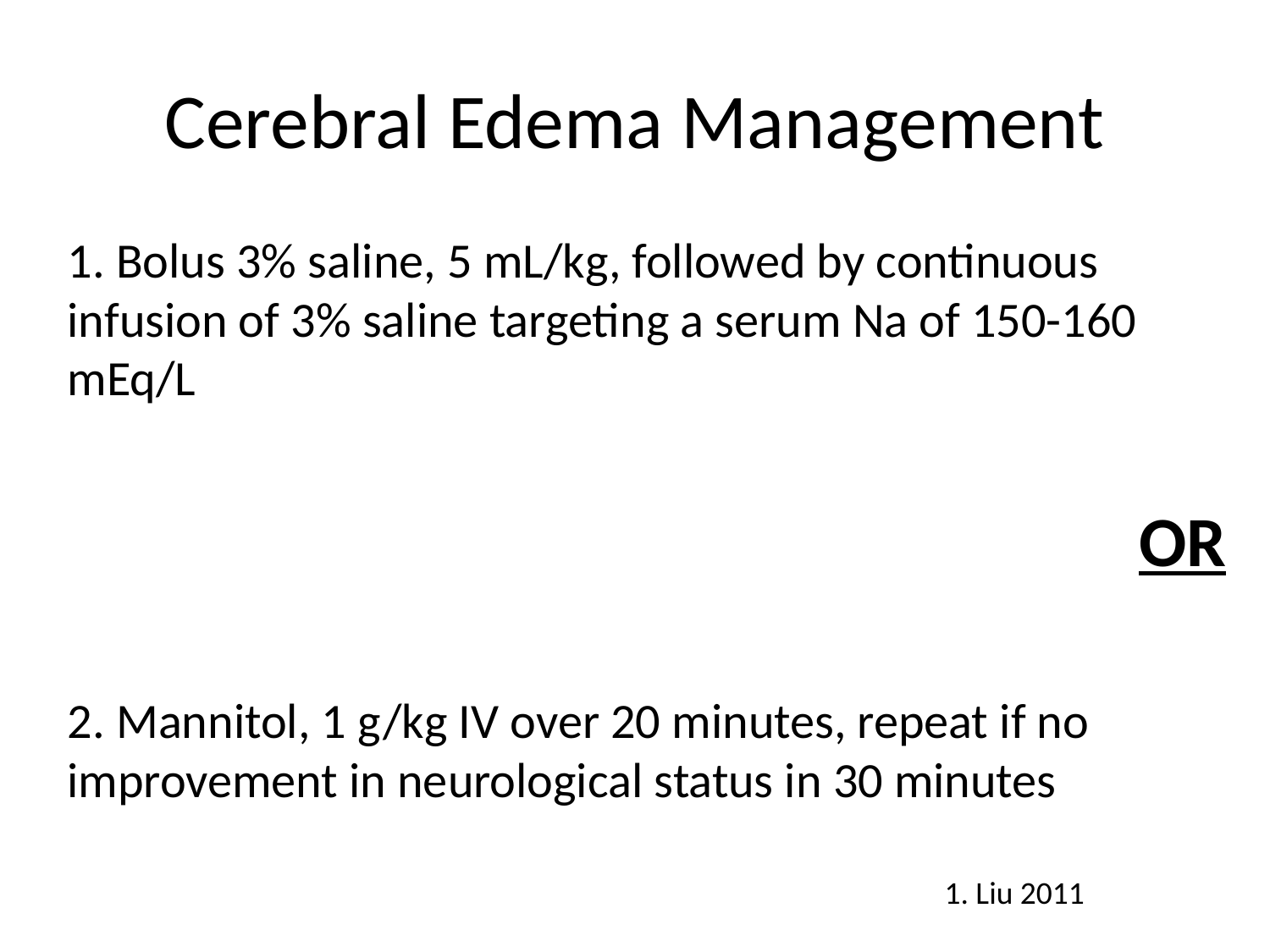

# Cerebral Edema Management
1. Bolus 3% saline, 5 mL/kg, followed by continuous infusion of 3% saline targeting a serum Na of 150-160 mEq/L
								OR
2. Mannitol, 1 g/kg IV over 20 minutes, repeat if no improvement in neurological status in 30 minutes
1. Liu 2011

## Slide 23
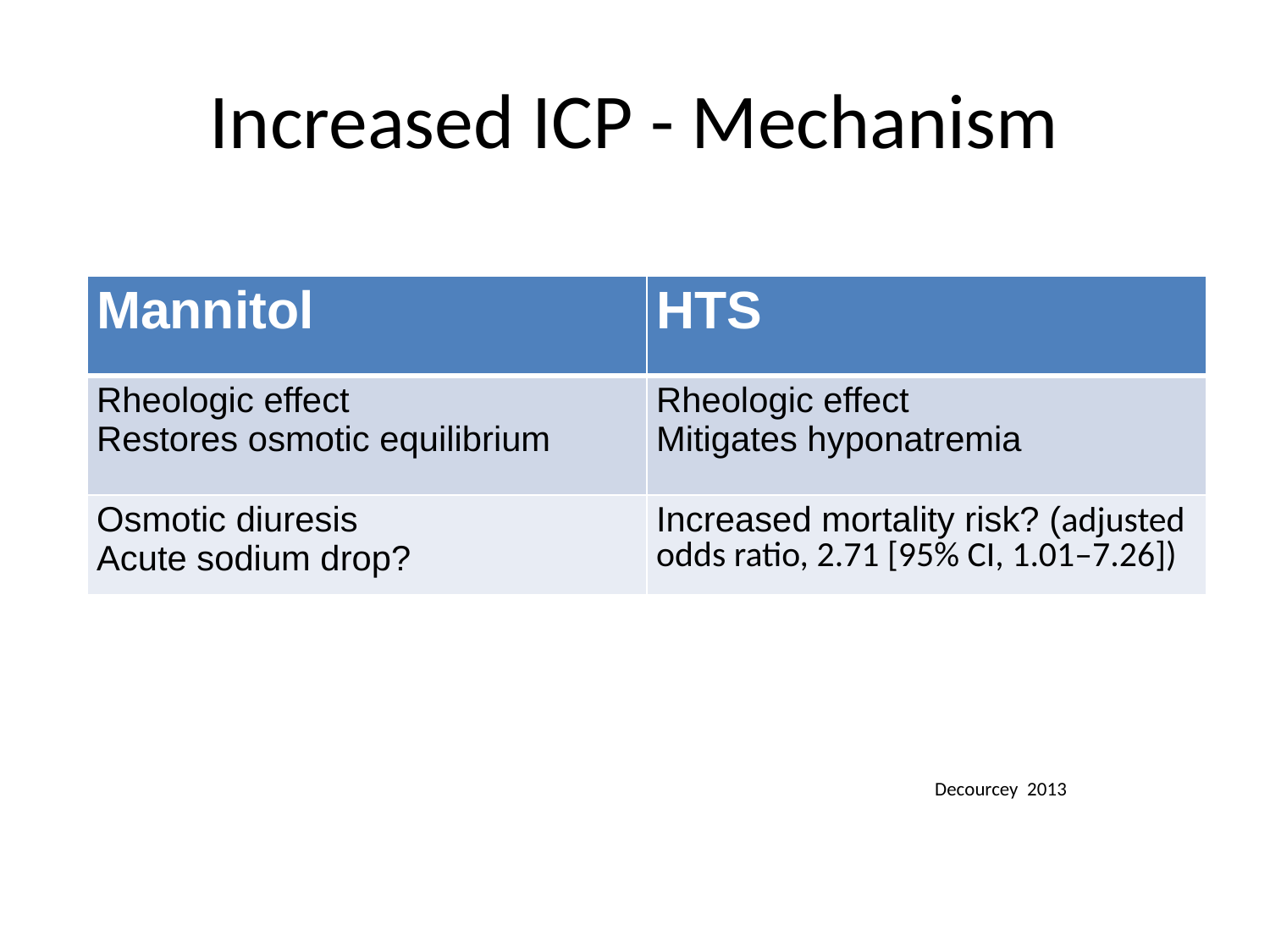

# Increased ICP - Mechanism
| Mannitol | HTS |
| --- | --- |
| Rheologic effect Restores osmotic equilibrium | Rheologic effect Mitigates hyponatremia |
| Osmotic diuresis Acute sodium drop? | Increased mortality risk? (adjusted odds ratio, 2.71 [95% CI, 1.01–7.26]) |
Decourcey 2013

## Slide 24
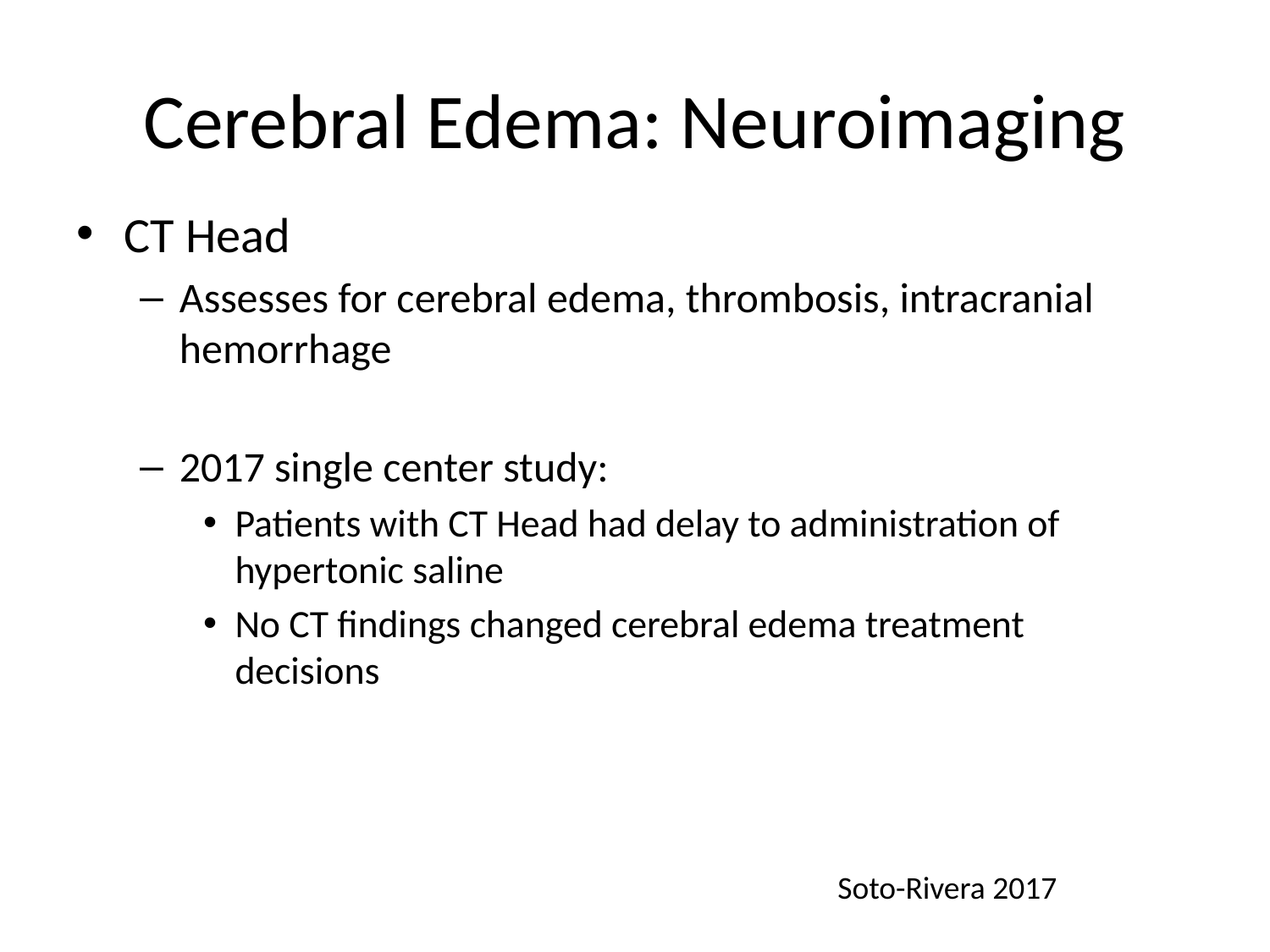

# Cerebral Edema: Neuroimaging
CT Head
Assesses for cerebral edema, thrombosis, intracranial hemorrhage
2017 single center study:
Patients with CT Head had delay to administration of hypertonic saline
No CT findings changed cerebral edema treatment decisions
Soto-Rivera 2017

## Slide 25
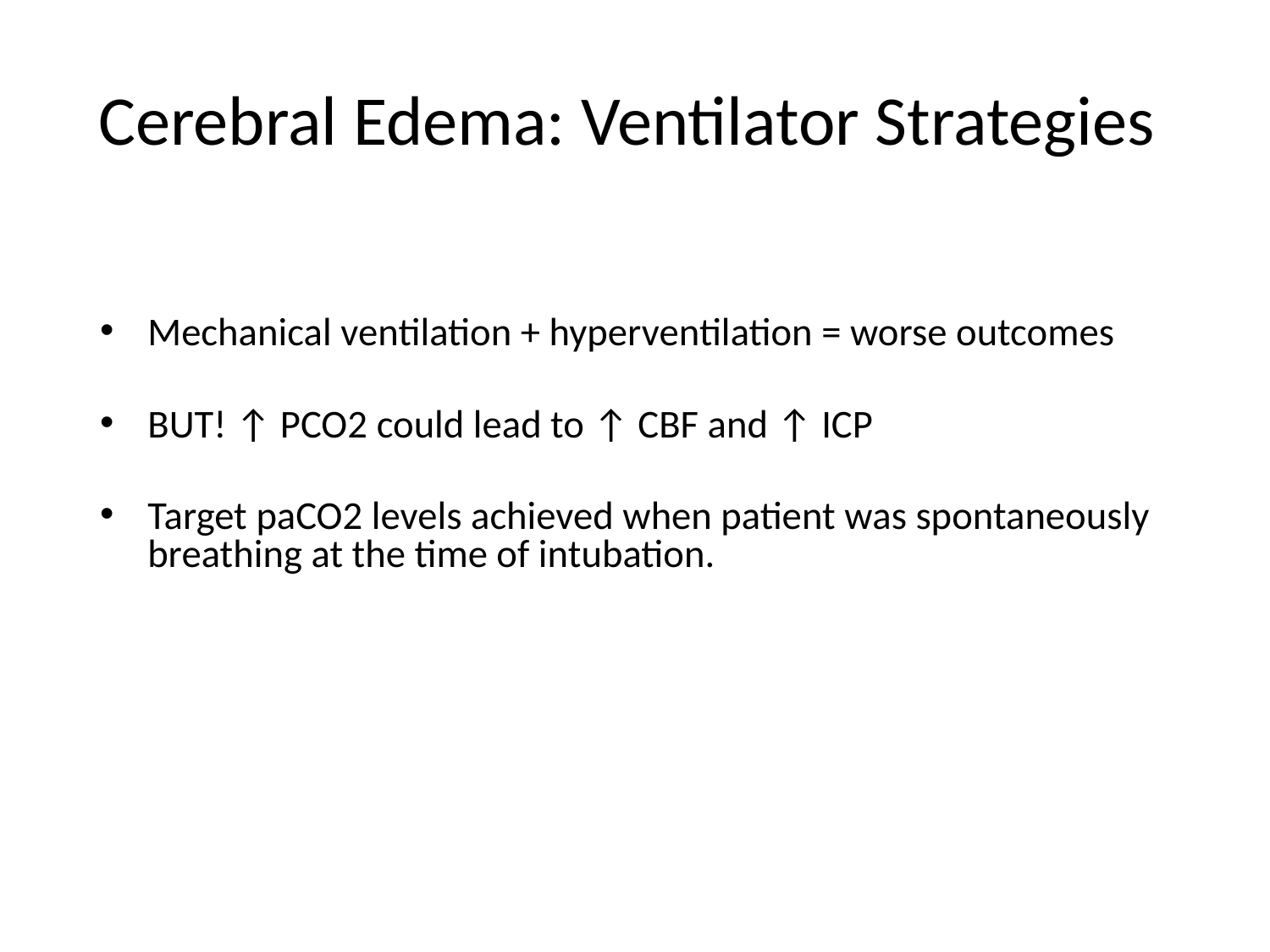

# Cerebral Edema: Ventilator Strategies
Mechanical ventilation + hyperventilation = worse outcomes
BUT! ↑ PCO2 could lead to ↑ CBF and ↑ ICP
Target paCO2 levels achieved when patient was spontaneously breathing at the time of intubation.

## Slide 26
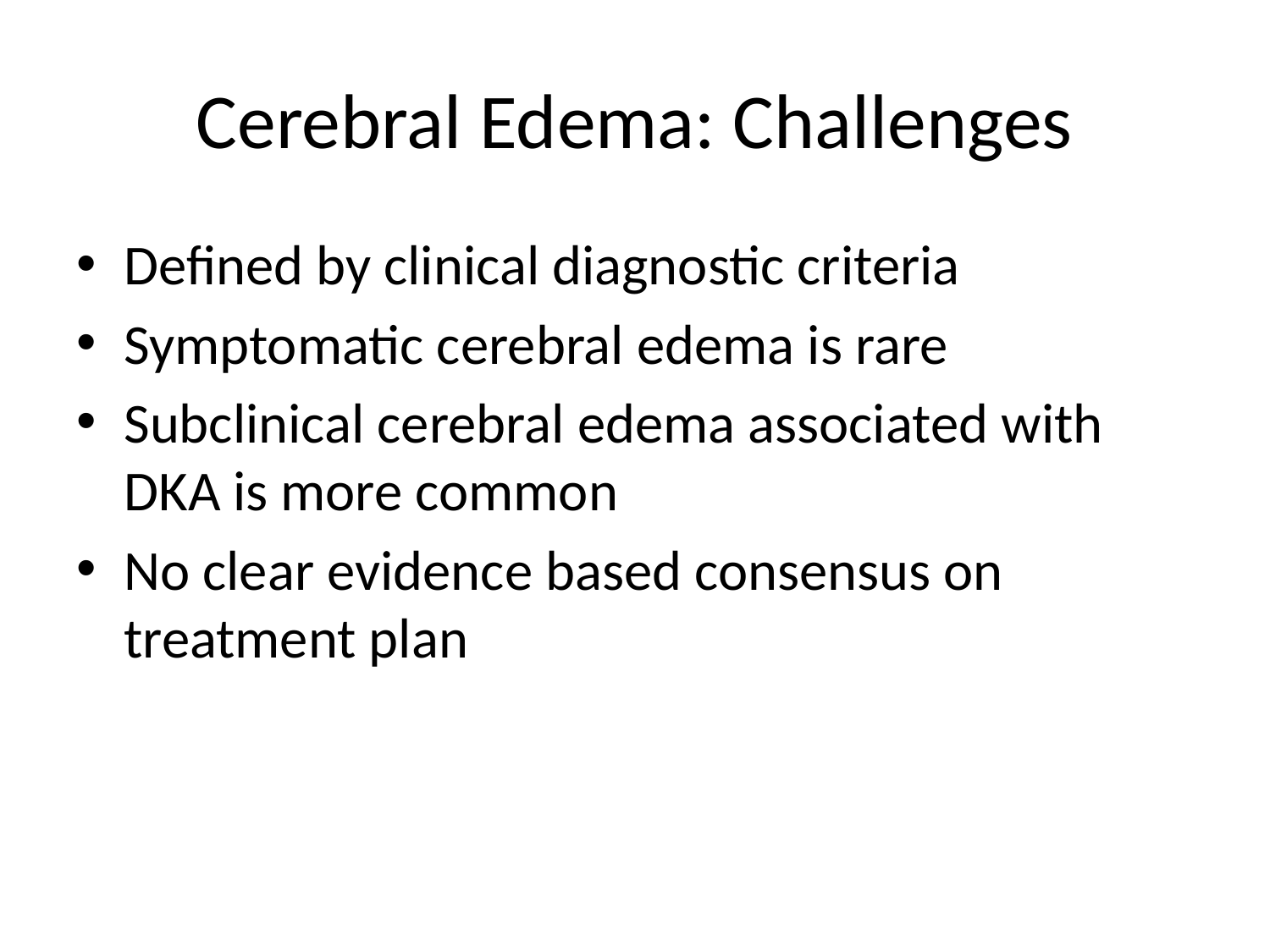

# Cerebral Edema: Challenges
Defined by clinical diagnostic criteria
Symptomatic cerebral edema is rare
Subclinical cerebral edema associated with DKA is more common
No clear evidence based consensus on treatment plan

## Slide 27
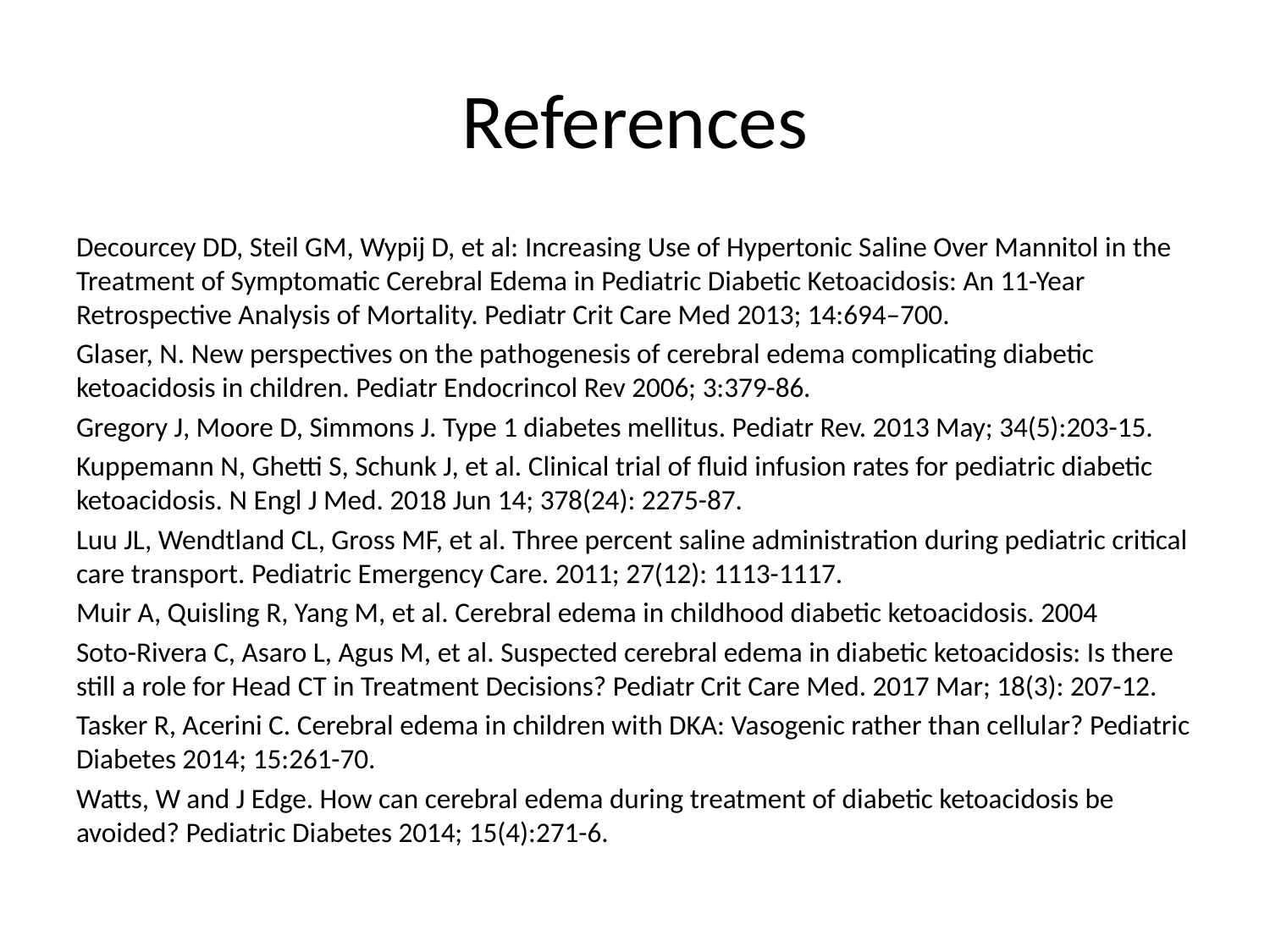

# References
Decourcey DD, Steil GM, Wypij D, et al: Increasing Use of Hypertonic Saline Over Mannitol in the Treatment of Symptomatic Cerebral Edema in Pediatric Diabetic Ketoacidosis: An 11-Year Retrospective Analysis of Mortality. Pediatr Crit Care Med 2013; 14:694–700.
Glaser, N. New perspectives on the pathogenesis of cerebral edema complicating diabetic ketoacidosis in children. Pediatr Endocrincol Rev 2006; 3:379-86.
Gregory J, Moore D, Simmons J. Type 1 diabetes mellitus. Pediatr Rev. 2013 May; 34(5):203-15.
Kuppemann N, Ghetti S, Schunk J, et al. Clinical trial of fluid infusion rates for pediatric diabetic ketoacidosis. N Engl J Med. 2018 Jun 14; 378(24): 2275-87.
Luu JL, Wendtland CL, Gross MF, et al. Three percent saline administration during pediatric critical care transport. Pediatric Emergency Care. 2011; 27(12): 1113-1117.
Muir A, Quisling R, Yang M, et al. Cerebral edema in childhood diabetic ketoacidosis. 2004
Soto-Rivera C, Asaro L, Agus M, et al. Suspected cerebral edema in diabetic ketoacidosis: Is there still a role for Head CT in Treatment Decisions? Pediatr Crit Care Med. 2017 Mar; 18(3): 207-12.
Tasker R, Acerini C. Cerebral edema in children with DKA: Vasogenic rather than cellular? Pediatric Diabetes 2014; 15:261-70.
Watts, W and J Edge. How can cerebral edema during treatment of diabetic ketoacidosis be avoided? Pediatric Diabetes 2014; 15(4):271-6.
