# Supplementary material for: Pediatric Emergency Medicine Didactics and Simulation (PEMDAS): Pediatric Diabetic Ketoacidosis
Source: MedEdPORTAL. 2021 Feb 17;17:11098. doi: 10.15766/mep_2374-8265.11098 (PMC7901255; doi:10.15766/mep_2374-8265.11098)
Supplement: Supplementary file 1 — Ped DKA Simulation Case.docxPed DKA Environmental Preparation.docxPed DKA Critical Actions.docxPed DKA ECG CXR Labs.docxPed DKA Debriefing Materials.docxPed DKA TeamSTEPPS Glossary.docxPed DKA Slides.pptxPed DKA Evaluation Form.docx [file mep_2374-8265.11098-s001.zip › H. Ped DKA Evaluation Form.docx]

**Appendix H: Pediatric DKA Simulation Evaluation Form**

**Instructor(s):** ___________________________________ **Date:**  ______________________

**Case Presented:** Pediatric DKA

**Circle one role**: attending/fellow/resident(peds, EM, FP)/medical student (MS3/MS4), other__________

|  | Strongly  Disagree | Disagree | Neutral | Agree | Strongly  Agree |
| --- | --- | --- | --- | --- | --- |
| 1. This case presented during the simulation is relevant to my work. | 1 | 2 | 3 | 4 | 5 |
| 1. The simulation case was realistic. | 1 | 2 | 3 | 4 | 5 |
| 1. This simulation case was effective in teaching basic resuscitation skills. | 1 | 2 | 3 | 4 | 5 |
| 1. The debrief promoted reflection and team discussion. | 1 | 2 | 3 | 4 | 5 |
| 1. The group discussion helped me develop and prioritize evaluation and management options for a child found to have new onset diabetes and DKA. | 1 | 2 | 3 | 4 | 5 |
| 1. The facilitators created a safe environment for discussion and exploration. | 1 | 2 | 3 | 4 | 5 |

After participating in this session, how confident are you in your ability to:

|  | Very Unconfident | Unconfident | Neutral | Confident | Very Confident |
| --- | --- | --- | --- | --- | --- |
| Demonstrate ability to assess and emergently manage airway, breathing and circulation | 1 | 2 | 3 | 4 | 5 |
| Formulate a list of possible diagnoses and prioritize elements of evaluation | 1 | 2 | 3 | 4 | 5 |
| Identify laboratory abnormalities diagnostic of DKA | 1 | 2 | 3 | 4 | 5 |
| Manage fluid resuscitation and insulin administration in a pediatric patient with new onset diabetes in DKA | 1 | 2 | 3 | 4 | 5 |
| Identify risks, signs and symptoms of cerebral edema associated with DKA | 1 | 2 | 3 | 4 | 5 |
| Construct a disposition plan after stabilization in the emergency department for a pediatric patient in DKA | 1 | 2 | 3 | 4 | 5 |
| Utilize effective team leadership, roles and communication strategies | 1 | 2 | 3 | 4 | 5 |

Can you list/describe 1 or more ways this session will change how you do your job?

How can we improve this simulation?

Additional Comments:
